# Developing a heritage BIM shared library for two case studies in Jordan’s heritage: The House of Art in Amman and the Qaqish House in the World Heritage City of As-Salt

**DOI:** 10.1186/s40494-022-00836-w

**Published:** 2022-12-12

**Authors:** Rania Aburamadan, Claudia Trillo, Victoria Andrea Cotella, Elena Di Perna, Chiko Ncube, Athena Moustaka, Chika Udeaja, Kwasi Gyau Baffour Awuah

**Affiliations:** 1 School of Architecture, Applied Science University, Al Arab St 21, Amman, Jordan; 2 Department of Civil and Structural Engineering, University of Bradford, Richmond Rd, Bradford, BD7 1DP UK; 3 Department of Architecture, University of Naples Federico II, Via Forno Vecchio 36, 80134 Naples, NA Italy; 4 Department of Civil Engineering, University of Salerno, Via Giovanni Paolo II, 132, 84084 Fisciano, SA Italy; 5 School of Built Environment, Oxford Brookes University, Headington Rd, Headington, Oxford, OX3 0BP UK; 6 School of Science, Engineering and the Environment, University of Salford, Newton Building, Crescent, Salford, M5 4NT UK; 7 Department of The Built Environment, London South Bank University, 103 Borough Rd, London, SE1 0AA UK

**Keywords:** BIM families, Shared library, Heritage dissemination, Digital technologies, Parametric objects, Jordan

## Abstract

International research is moving towards the development of BIM (Building Information Modelling) libraries applied to the built heritage where one of the main issues to be addressed is the modelling of complex or unique shapes that represent the specific construction components of every single asset. This perspective addresses the generation of parametric families of representative architectural geometry in the context of the management and representation of a building of heritage value. Jordan’s architectural heritage has gone through a long period of evolution and development: the result is a mixture of influences and traditions, making a great stride to conserve its buildings and Historical Heritage but has never adopted advanced digital technologies such as Building Information Modelling. In this framework, the present research aims to bridge the gap in Jordan by applying digital technologies to support heritage conservation plans by creating a 3D library of BIM objects related to typical elements of Jordanian and Arabic architecture, specifically in two cases of study. Co-production and collaboration with diverse stakeholders were central to the development of the methodology and design of the research.As a result, the first open-access HBIM shared library of historical features of Jordanian built heritage will be consolidated; this is crucial because it will set a precedent for the further documentation and conservation of the heritage of traditional cities in Jordan, MENA countries and internationally by promoting social cohesion, economic and technological development, tourism and the awareness of Jordan’s cultural heritage.

## Introduction

Jordan is a MENA—Middle East/North Africa—country, characterised by serious problems regarding the conservation of built heritage, both archaeological and historical [1]. Culture and heritage in Jordan are no longer the domain of specialised experts in archaeology. In this context, the role of digital technologies has evolved in heritage conservation evolving into a medium for cultural identity and cross-cultural communication as well as a resource for tourism, economic and social development of local communities and regions in Jordan [1]. Architectural documentation has a fundamental role in conserving the built heritage since it is supportive of conservation interventions. The significance of this methodology lies in the fact that, over time, there is the possibility that the physical documentation may be fragmented and, therefore, be lost.

Introducing digital technologies, such as Heritage Building Information Modelling (HBIM), in the documentation of the built heritage leads to the possibility of creating 3D parametric models that can be used as efficient communication tools, enclosing historical information, conservation status, data on the materials and construction techniques used [2]. Indeed, HBIM offers the possibility to integrate multiple layers of information and to link across industry, community and higher education with a flexibility and timeliness that traditional techniques such as paper-based drawings may not allow [3]. The connection between these layers allows for fewer discrepancies and errors that may occur when works are not completed in an integrated way, creating an integral base in which to access all the different data information about a particular heritage asset is also complex and interconnected with other actors like engineers, architects, economists, historians, and more, who work in a more collaborative and shared approach, cooperating with a common purpose.

In the Jordanian construction sector, BIM is a recent methodology although it is widely used in the rest of the world. In fact, a study investigating the status of HBIM adopted in the heritage and construction sector in Jordan has not yet been carried out, as most Jordanian construction and conservation is mainly based on 2D representation [4].

Currently, a shared library for historical Jordanian elements does not exist, hence why HBIM can help to create a library of objects related to the traditional architectural heritage in Jordan, objects that can be used by every entity involved in the intervention on the built heritage [4]. HBIM’s most important feature is the integration of data allowing for interdisciplinary decisions to be made based on models created and shared using BIM tools [5].

The present research is based on the project Herit-IT Conservation in Jordan [6], funded by the Royal Academy of Engineering, 2019–2022, aiming to use an HBIM approach for the conservation of Jordanian architectural heritage, through the generation of a library of architectural parametric elements starting from their survey, accessible by every practitioner operating in the AECO (Architectural, Engineering, Constructions and Operations) sector in Jordan and the rest of the Arabic world. The main platform of the Herit-IT Jordan project (https://herititjordan.com/) and related library (https://hitj.centroictbc.unisa.it/) include a range of digital outcomes for the two pilot heritage buildings of the Jordanian House of Art in Amman and Qaqish House in As-Salt, whose potential applications includes virtual tours, point clouds, H-BIM model of the building, and H-BIM families of the architectural features.

The following sections of the document aim specifically at proving the adopted procedure. Section “Literature review: HBIM libraries and digital platforms” presents a literature review regarding HBIM libraries and digital platforms. Section “Methodology” describes the methodological structure, followed by Section “Digitising Jordan’s Historic Houses: Case study of House of Art, Amman and Qaqish House, As-Salt” which presents the case studies. Sections “Developing an open-access HBIM shared library” and “Online 3D BIM library” explain the development of 3D objects and their dissemination on the digital platform. Section “Final outputs and discussion” reports the results obtained and, finally, Section “Conclusion” closes this paper with the necessary reflections and conclusions.

## Literature review: HBIM libraries and digital platforms

Currently, in order to rigorously represent the architectural geometry and the constructive solutions adopted in historic buildings, there is a need to create specific parametric object families due to the fact that libraries available in BIM-based modelling systems are currently very limited and mostly adapted to new constructions. With this background, the research field is moving towards the development of HBIM libraries regarding different architectonical elements and Open-Source platforms for data sharing.

In the first instance, simplified parametric models suitable for industrial elements and modern architecture—specifically wooden vaults and bean floors—were developed to build an abacus of local constructive elements starting with a TLS survey and exporting the results to IFC format [7]. The semantic classification of the component and sub-components was carried out in three related categories: Definition of the macro-family’s “component” of structural elements, definition of hierarchical aggregation of different object elements composing the object family (structural elements, non-structural elements, and decorative layers) and finally the definition of the material of each object element.

A methodological multi-level workflow to develop an HBIM library for the vaulted systems of the Palazzo Magio Grasselli in Italy was applied, with the particularity of exporting the models into the GIS environment [8] by integrating different types of data in one dynamic model, and requiring the management of different Levels of Detail (LOD) to establish a correct hierarchy of information between BIM and GIS. The interaction of different software such as AutoCAD, Rhinoceros and Revit was essential in the different workflow phases.

Algorithmic modelling by using Dynamo offers several possibilities to design complex construction elements starting from the reconstruction of different ancient columns in several case studies. In a first instance, a workflow which develops library objects from 3D CAD primitives using architectural rules to construct parametric representations of classical architectural elements was studied [4]. In addition, a Doric Colum of the Temple of Hephaestus in the Ancient Athens Agora was modelled. After that, to parametrize the geometry, the real point cloud data were inserted into Revit and the dimensions of the modelled column were suitably modified to best fit the point cloud [9]. Furthermore, a whole new series of workflows is created that foresee within them the insertion of visual programming tools as an intermediate step or a substitute for those of classical methodologies applying them to a set of architectural columns belonging to the Italian Cultural Heritage [10].

On the other hand, there is a large number of studies on the development of collaborative Open-Source BIM platforms to ensure access, visualization and download of IFC files. It was developed a first Cloud-based system to provide a web-based service to manage BIM combining different technologies, such as Cloud computing with the Nextcloud software equipped with plugins “BIM Surfer” and BIMviews [11]. Furthermore, consistent solutions include BIMServer.center (https://bimserver.center/en/), a system that, besides managing, sharing and updating projects in the cloud, allows the user to access the BIM project in Virtual Reality (VR) and Augmented Reality (AR) environments, unlocking a new level of accessibility and immersion; another consistent solution is BIMServer (https://github.com/opensourceBIM/BIMserver), an open-source web platform (via browser) reachable through a JavaScript server app that allows to manage and share the BIM project [12].

## Methodology

Investigation and documentation of the built heritage are essential for its conservation and subsequent dissemination [13]. To reach this goal, several and constant interventions are conducted under an organisational management process, which requires a specialised methodological basis to address the complexity characterising historic buildings. Herit-IT Jordan aimed to bridge the divide in Jordan by applying digital technologies to support heritage conservation plans. Co-production and collaboration with diverse stakeholders were central to the development of the methodology and design of the research. The tri-national team engaged early on with national and local stakeholders in two main cities: (1) Capital City, Amman; and (2) World Heritage City of As-Salt.

Despite the focus on the application of digital technologies to heritage, the fundamental goal was to influence and promote social cohesion, economic and technological development, tourism, and awareness of Jordanian cultural heritage. Early discussions with local stakeholders in the cities led to the co-identification of local vernacular architectural houses in each city that served as an ideal for digitisation and application of HBIM. In the city of Amman, the research team in collaboration with Greater Amman Municipality selected the Jordanian House of Art. In the city of As-Salt, the As-Salt Municipality and the research team selected the Qaqish House. National stakeholders such as the Jordanian Tourism Board and the National Heritage Council played key roles throughout the research project.

The developed methodological process is based on the problem of the fragmentation and inaccessibility of the information by all the entities of the interdisciplinary management process. The process is divided into three main global phases: the first phase focused on the study of the state of the art of conservation of Jordanian architecture and the implementation of digital technologies. The second phase focused on the knowledge of the object of study on which to intervene, the House of Art and the Qaqish House: this phase includes the digital survey and the resulting high-precision point cloud that was later used as the basis for the geometric knowledge of the object and the features of the elements composing it. The last phase includes the 3D modelling of the elements that complete the building, developing Revit families starting from both the point cloud and the virtual tour, with the addition of parameters and texture information, and finally the development of the digital platform.

## Digitising Jordan’s Historic Houses: case study of House of Art, Amman and Qaqish House, As-Salt

**Fig. 1:**
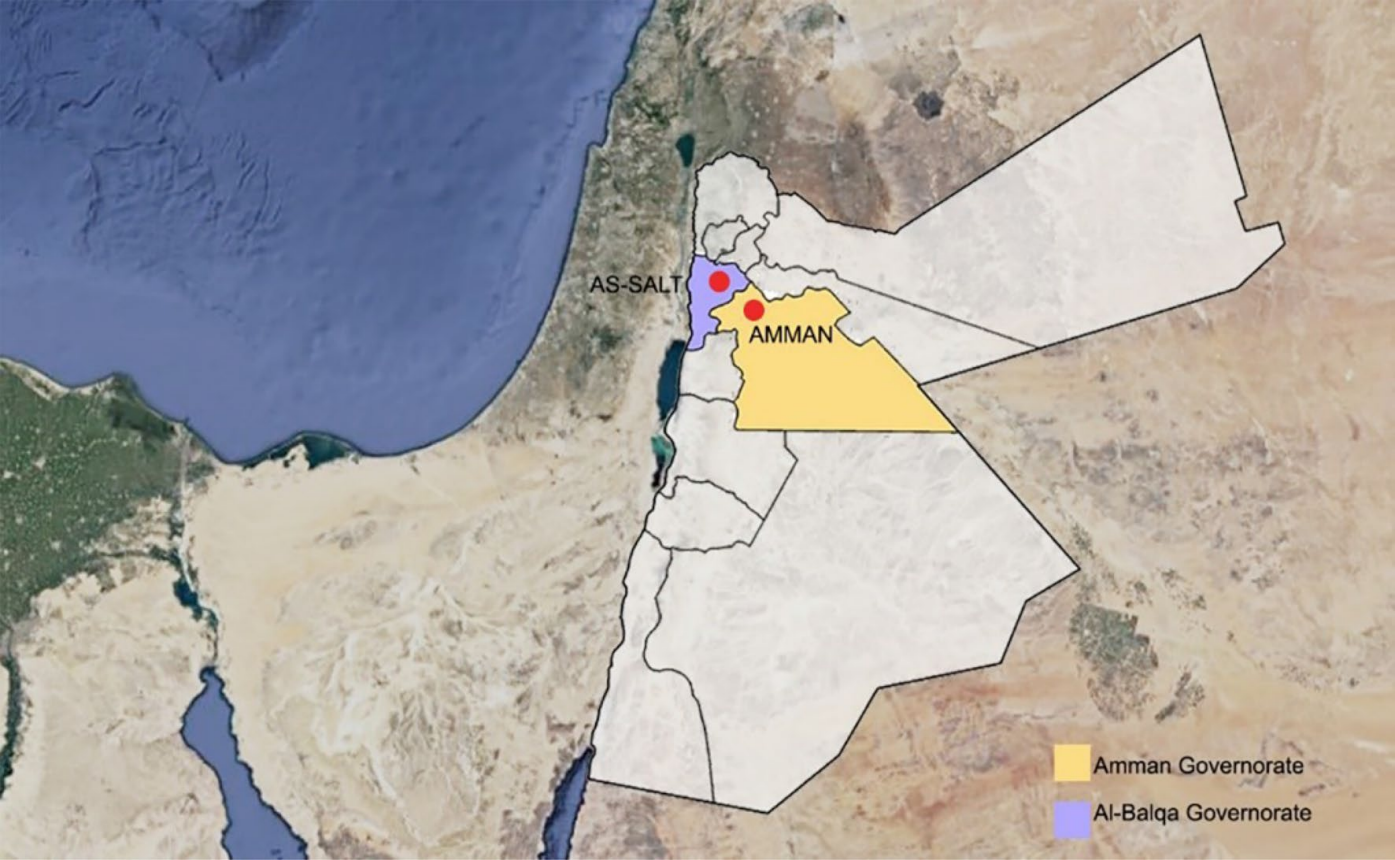
Overview map of Jordan highlighting the cities of As-Salt and Amman

Conservation legislation is under the responsibility of the Ministry of Tourism and Antiquities. The first act of legislation in Jordan protecting cultural heritage is the Law of Antiquities No. 21 for the year 1988 and No. 23 for the year 2004: it is evident how this leaves unprotected significant layers of heritage from the Ottoman period onwards. The case studies are a clear example of it: the Qaqish House in the city of As-Salt and the House of Art, in Amman, built respectively in 1866 and in 1923 (Fig. 1).

The city of As-Salt, capital of the Al-Balqa Governorate, was added to the UNESCO World Heritage list in 2021 as ‘the city of tolerance and civilised hospitality’: during the last 60 years of the Ottoman period, Muslims and Christians developed traditions of hospitality evidenced in Madafas (guest houses, known as Dawaween) and the social welfare system. The city’s urban core contains approximately 650 significant historic buildings; four of them, including one of the oldest notable residences in Al-Salt, the Qaqish House, are owned by the Ministry of Tourism and Antiquities and the Greater Municipality of As-Salt. Currently, The Qaqish House serves as the office of As-Salt’s City Development Project (ASCOP2016) [14].

On the other hand, the city of Amman, the capital of the country, hosts the Jordanian House of Art, one of the first buildings that started the gradual transformation of Amman from a village to a city of its oldest and most bustling streets; it is located on Prince Muhammad Street, which is one of the oldest and most bustling streets of the capital. The House of Art has particular decorative elements: the floor tiles, for example, were brought from Palestine to Jordan, indicating the important connection between Jordan and Palestine. After a long period in which the house has held different roles, it was inaugurated as an art museum in May 2002 by Queen Rania Al Abdullah, during the celebration of the nomination of Amman as Arab Culture Capital, showing the importance of this building for Jordan and its heritage; this happened through the efforts of entities such as the Jordanian Ministry of Culture, the Jordan River Foundation, the Association of Finest Technicians, the Department of Public Antiquities and the Jordanian Music Academy,.

### Jordanian House of Art, Amman

**Fig. 2:**
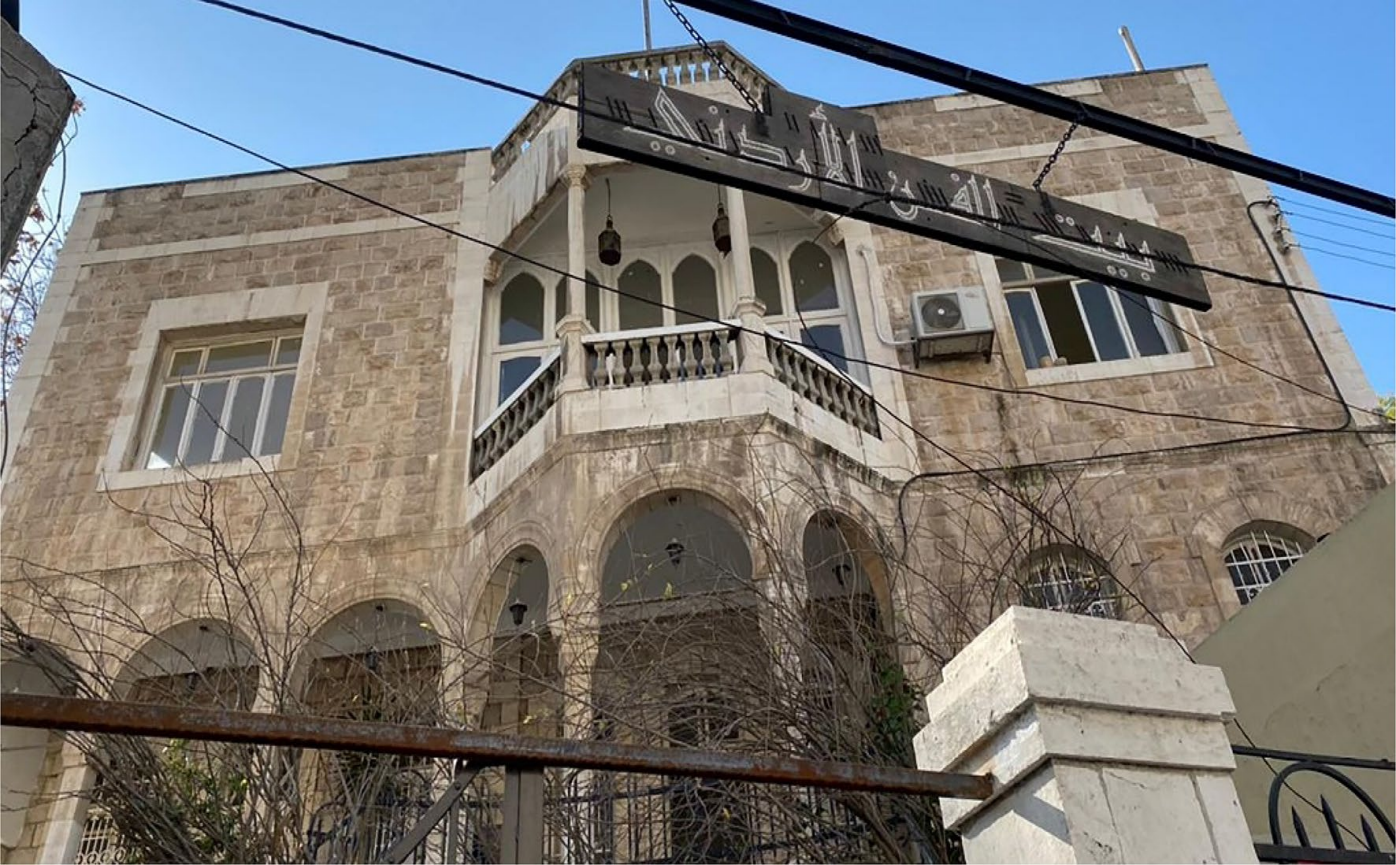
Main façade of the House of Art

The Jordanian House of Art (Fig. 2)—in Arabic “Beit Al-Fann”—is an original Ammani architectural building, characterising the social, cultural, and political timeline of the city, holding historical, artistic, and cultural value [15]. This building is one of the first buildings that started the gradual transformation of Amman from a village to a city [16].

The house is owned by a rich and military figure family, the Al Sukhun family. It is located on the outskirts of Downtown Amman, in the neighbourhood of Al Rjoum, in one of the oldest and most bustling streets of the capital, Prince Muhammad Street [17]. Anciently, this street was among Amman’s first commercial areas, representing a commercial valley between residential hills [15].

It is a house where privacy is present since its entrance is set back from street level. The orientation of the house did not follow the street line but was built dynamically oriented towards a different angle, in such a way to ensure privacy and to have a wider entrance preceding the construction. At the end of the stairs, the visitor arrives at a large entrance in which stone arches of beautiful design rise [18].

**Fig. 3:**
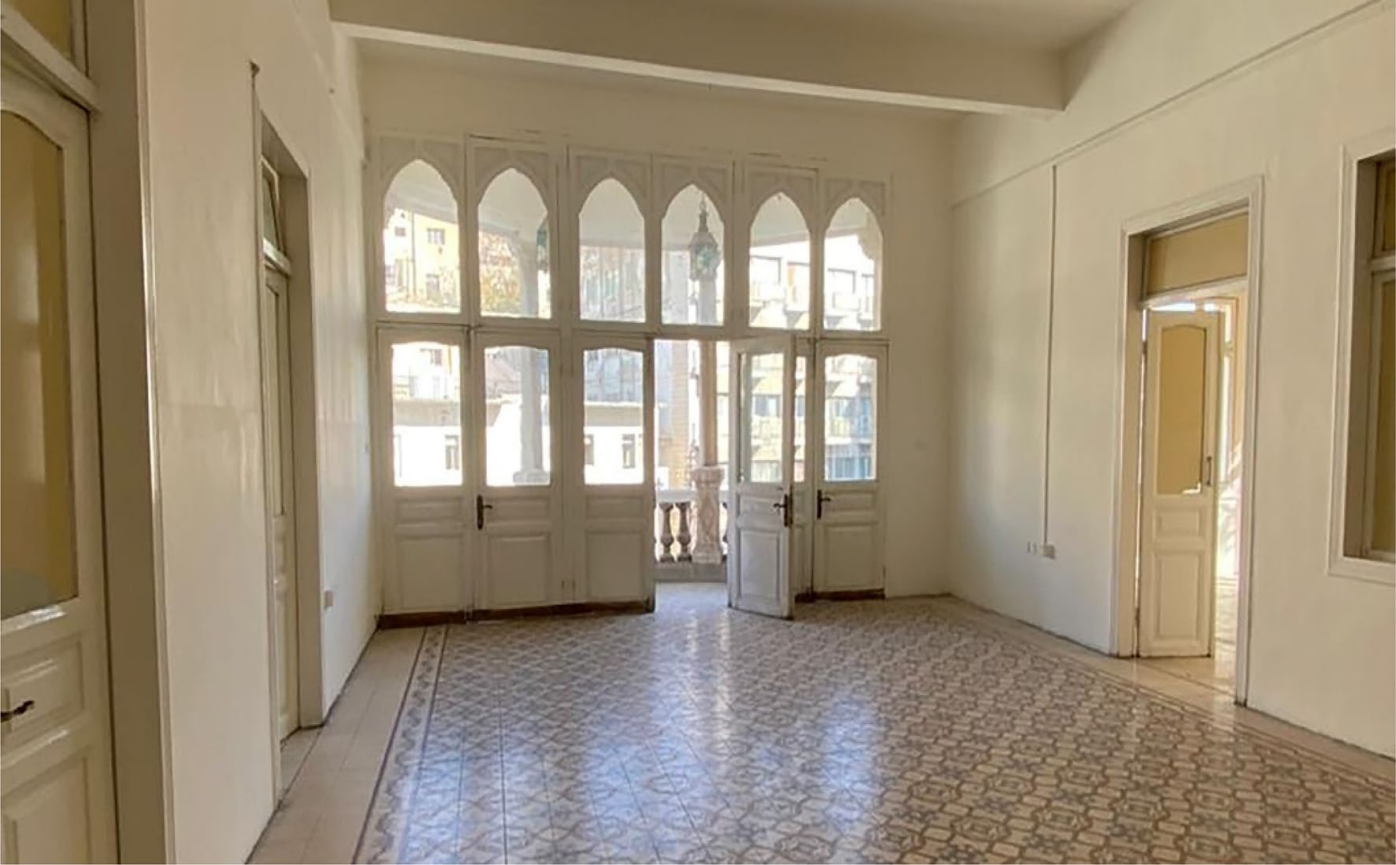
Interior space of the House of Art

The “Beit Al-Fann”, (Fig. 3) has not always been in the form it can be seen today. It was designed by the Palestinian architect Fawwaz Al-Muhanna consisting of two storeys. The work was split into two stages, the first one included the construction of the ground floor, which took around three years between 1923 and 1926; the first floor was added in 1937.

Built with a total area of 1300 square meters, a significant percentage of which was made of stones brought from Palestine representing a mix of eastern and western influences [19]. The stones needed to complete the construction were collected directly from quarries in the areas near the capital. As for the roof of the house, the sand for its construction was brought on camels by the stream of Oman. Furthermore, the tiles arranged in the interior of the building differ from one room to the other in pattern and colour.

The construction's most eminent attribute is the interesting relationship it creates between the vegetation and the rising building elements, through which the landscape does not feel forced or artificial, but part of the spatial identity distinctive of the territory.

Over the years the house has held different roles, as the home of the first commander of the Jordanian Desert Force, Ahed Al-Sukhun; Al-Zahraa School for girls; it finally opened in 2002, after 7 years of restoration, with the new and current name of “The Jordanian House of Art”, enclosing an art museum, a theatre, and a cultural centre that preserves the memory of past generations and shows the authentic Jordanian Heritage [15, 20].

### Qaqish House, World Heritage City, As-Salt

**Fig. 4:**
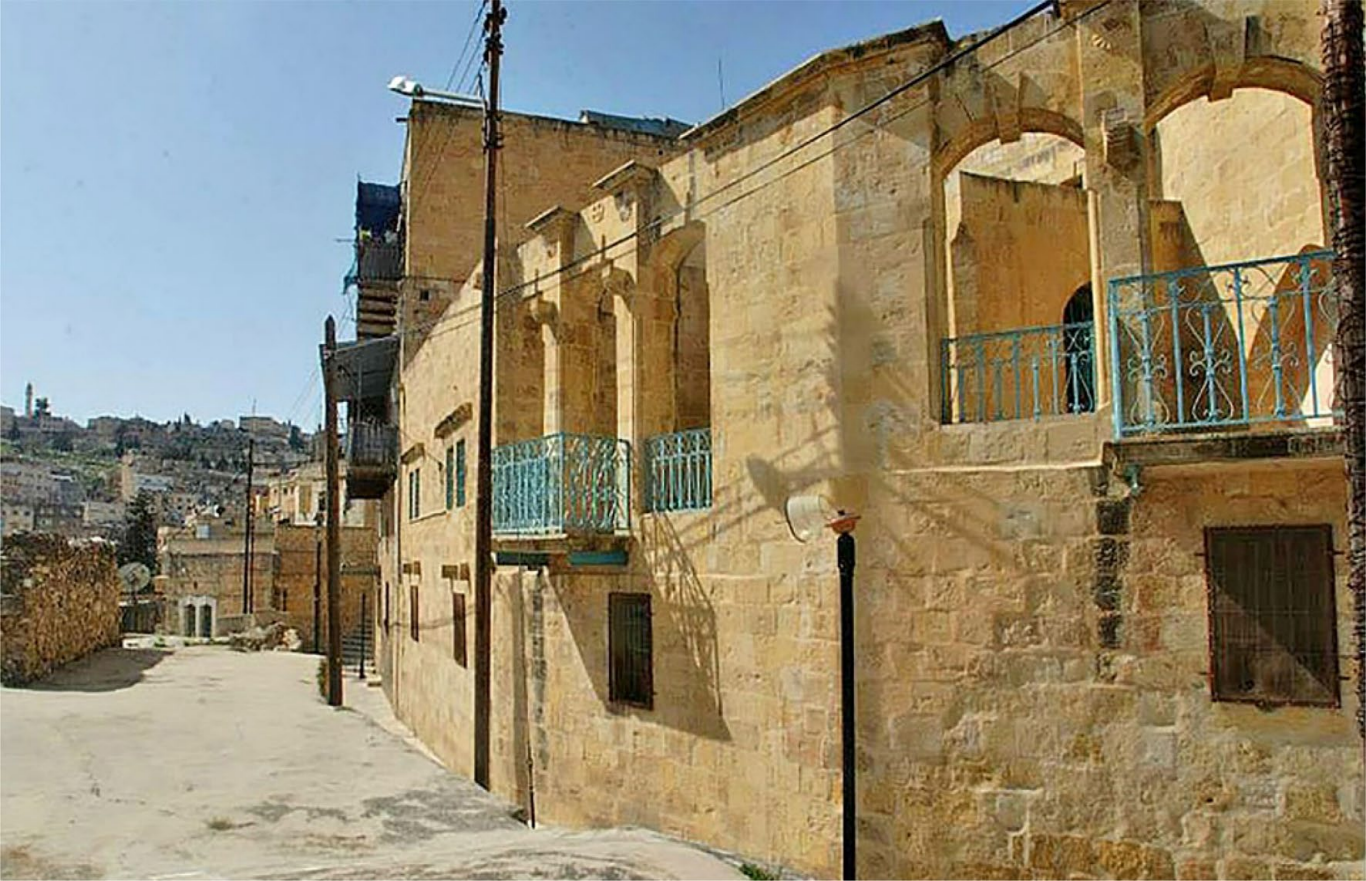
Outdoor terrace on the first floor of the Qaqish House, As-Salt

The Qaqish House (Fig. 4)—or Beit Qaqish, Arabic for “The House of the Qaqish”—is located in the Al Wal’a area, in Downtown As-Salt [21], and it is connected to the north of Al-Khader Street through the main stairway. The house is one of the oldest notable residences of As-Salt City, in west-central Jordan: it was built during the urbanisation period that characterized the city during the Ottoman empire, when elite merchants took As-Salt as a safe city for trade expansion to the east [22].

**Fig. 5:**
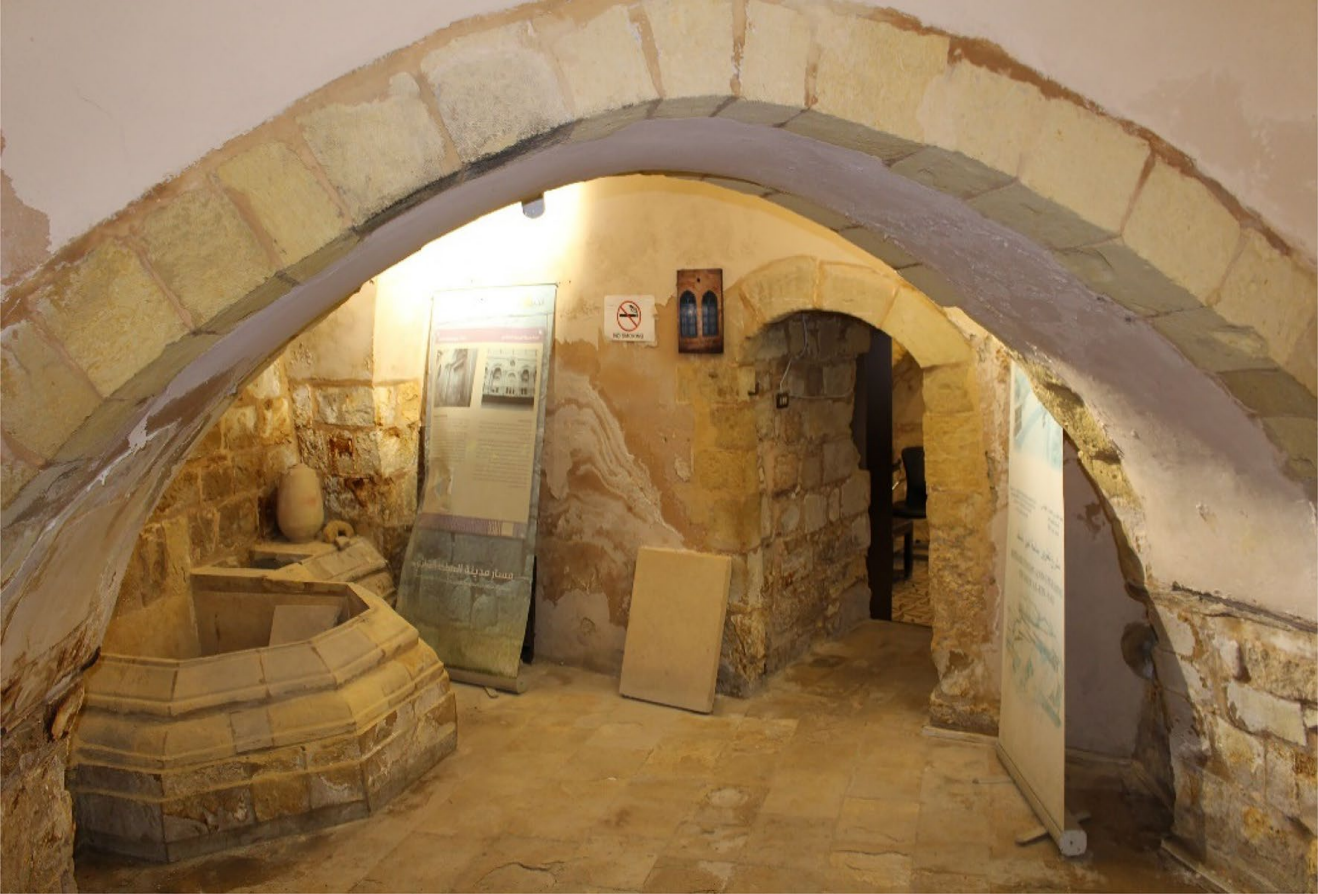
Cross-vaulted iwan in the Qaqish House

Its construction started in 1866 to end 39 years later, in 1905, upon the request of the Qaqish family. It can be described as an extended “peasant” family house, then transformed into a merchant house, with a cross-vaulted iwan (Fig. 5) and a double terrace decorated by a false front wall with openings.

The building has a floor area of 300 square meters. It is orientated to the east, characteristic of the old cathedrals. It is composed of two floors: the entrance consists of three halls big enough to accommodate pilgrims and religious leaders who used to visit the house from the nearby churches. In the hall closer to the entrance, it can be found a fountain that was used to bring water to the horses that were placed in two halls to the right of the entrance—the walls of these halls are smooth and curved to allow horses to move freely and reduce obstacles. This led to having the rooms divided into two floors, in order to have privacy and segregation between public and private, between men and guests in the common area on the ground floor, and women and family on the first floor in the back. In order to increase privacy, on the first floor there is an outdoor terrace backed up from street level, in such a way to have a buffer zone between the exterior and the interior of the house.

In 1989 the house underwent restoration, addressed to secure the house and improve its aesthetics. Starting from 2006, the house has been donated to As-Salt Municipality, which converted it into the office of the As-Salt City Development Project.

## Developing an open-access HBIM shared library

### Digital survey

Data acquisition has always been of crucial importance in the documentation of tangible Cultural Heritage. Architectural surveying is an evolving field that has changed significantly during the last decades due to technological advances in the field of 3D data acquisition [23]. Currently, the use of digital technologies such as LiDAR (Light Detection and Ranging), Aerial Photogrammetry and Wearable Mobile Laser System (WMLS) are leading in the field of surveying.

Among the LiDAR technologies, Terrestrial Laser Scanners (TLS) can have a wide range of applications in the documentation of cultural heritage, from small objects to large complex buildings, due to real-time data acquisition on a real scale, high accuracy and high speed and the production of a large number of points [24]. In the present case studies, the instrumentation used to carry out the survey consist of the Leica RTC360, a TLS by Leica GeoSystems, [25]. This instrument is the smallest and lightest imaging laser scanner available on the market (Table 1) and it is composed of a spherical 3-camera system of 15 Mpixel and a thermography panorama sensor system.

**Table 1:** BLK360 product specifications by Leyca Geosystems

Dimensions	Height: 165 mm/Diameter: 100 mm
Weight	1 kg
Laser class	Class 1 (in accordance with IEC 60825-1:2014)
Scanning range	min. 0.6—up to 60 m
Point measurement rate	Up to 360’000 pts/sec
Measurement speed	< 3 min for complete full-dome scan, spherical image & thermal image

**Fig. 6:**
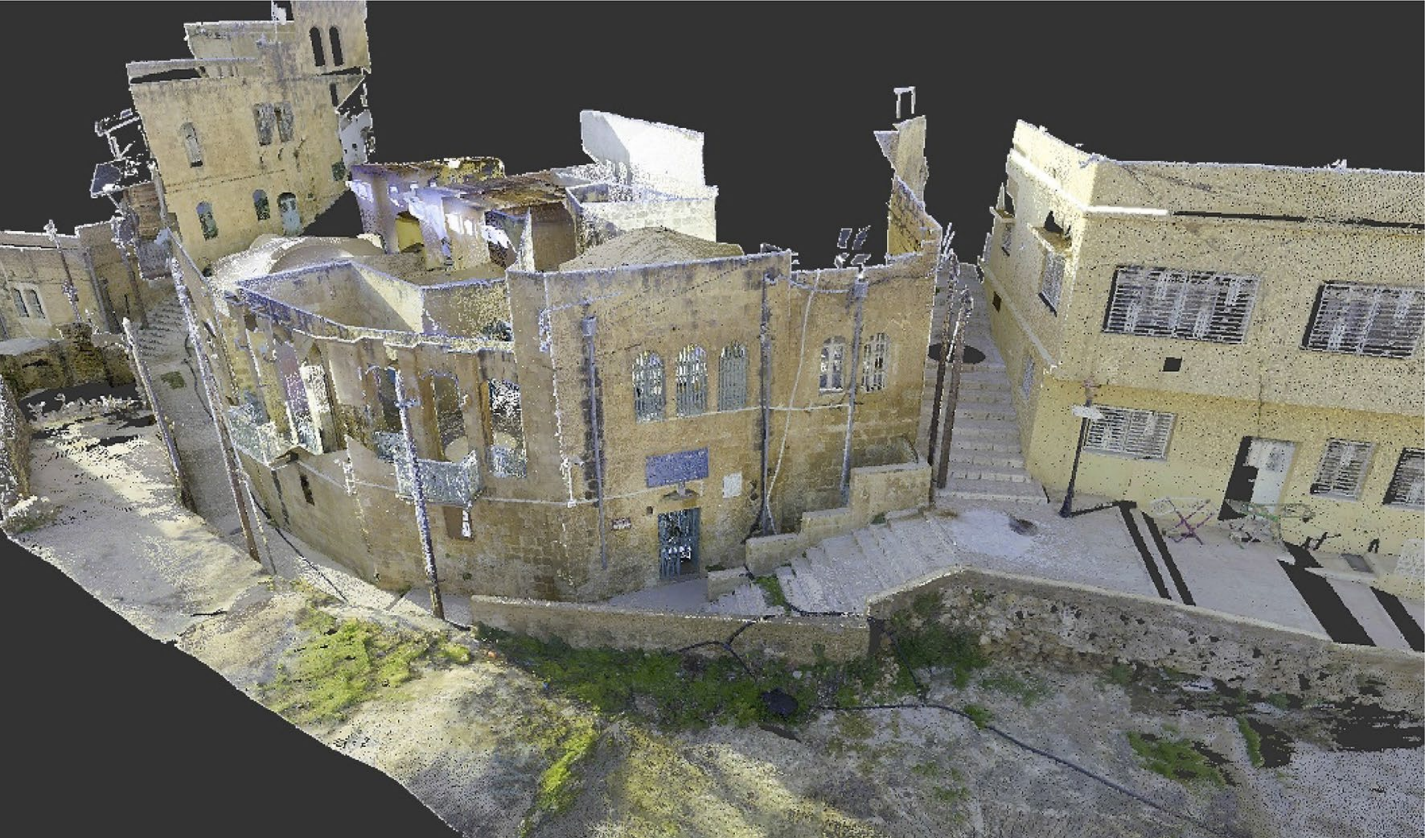
Detail of the point cloud of the Qaqish House

The first step of survey design should first define TLS station locations to ensure the complete coverage of the object at the required spatial resolution. In both cases, considering the complexity of the interior space, the instrument was set to have a resolution of 6 mm in 10 m which gathered a total of 47 internal and external scans from the House of Art and 56 from the Qaqish House (Fig. 6).

The second step of data processing has been done with the Leica Cyclone software: what Cyclone does, using the Iterative Closest Point algorithm, is to look for all the possible overlaps between the scans; for each overlap, the program chooses the best matching point pairs between the two scans. As the last thing, the program runs a minimization among these matching point pairs of all the connections, minimizing the global error [26]. From Cyclone Register 360 it is easy to export the data into an LGS or RCP file: these files will be manipulated on Autodesk.

The point cloud thus obtained, in this case from both houses, must then be uploaded on the software Recap by Autodesk, in order to cancel the noise in the scan: noise includes furniture, interference of people, and vegetation. This RCP file, once saved, can be opened with the software Revit to generate the three-dimensional model.

**Fig. 7:**
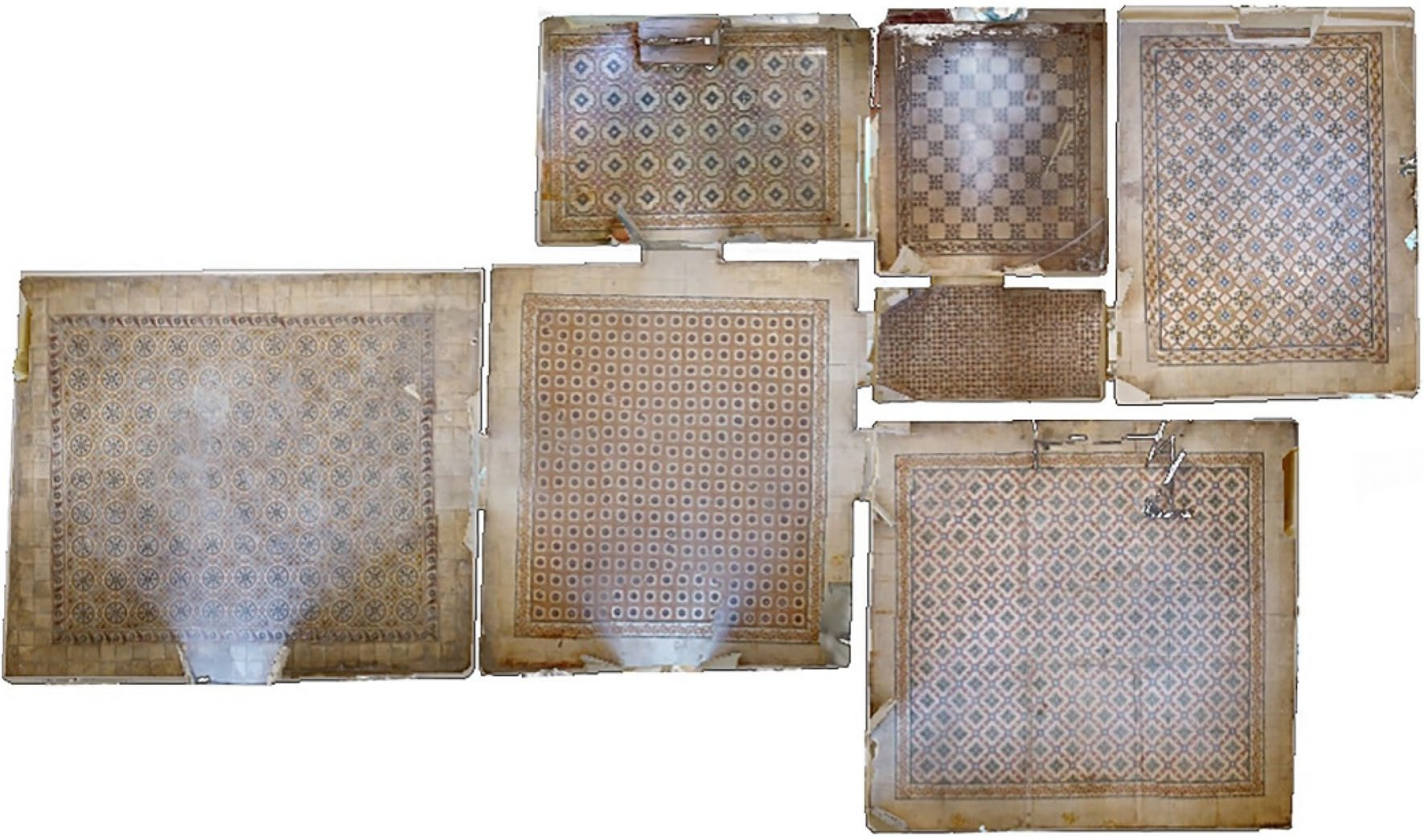
Detail of the different tiles used in the House of Art, as a result of the digital survey

On the other hand, also from the digital survey is possible to acquire the 360° spherical photos and videos that will be used in the development of the virtual tour and to appreciate the quality of the materials and geometry (Fig. 7). In fact, the real dimensions of the modelled objects will match exactly the information derived from the Virtual Tour and the Point Cloud.

### Generation of the BIM objects

In the present research, the methodology chosen is the Scan-to-BIM approach, a reverse modelling technique that uses digital sensing technologies to obtain point clouds that become the basis for BIM modelling oriented to existing buildings. Since Jordanian Architecture has a peculiar morphology, the project does not present standard components that may already be found in the Revit families’ catalogue. These elements need to be modelled from scratch with the aim of configuring the corresponding parameters. To achieve this goal, a four-step workflow was established: a digital survey previously explained, a recognition-object phase, decision making and finally the re-design.

**Fig. 8:**
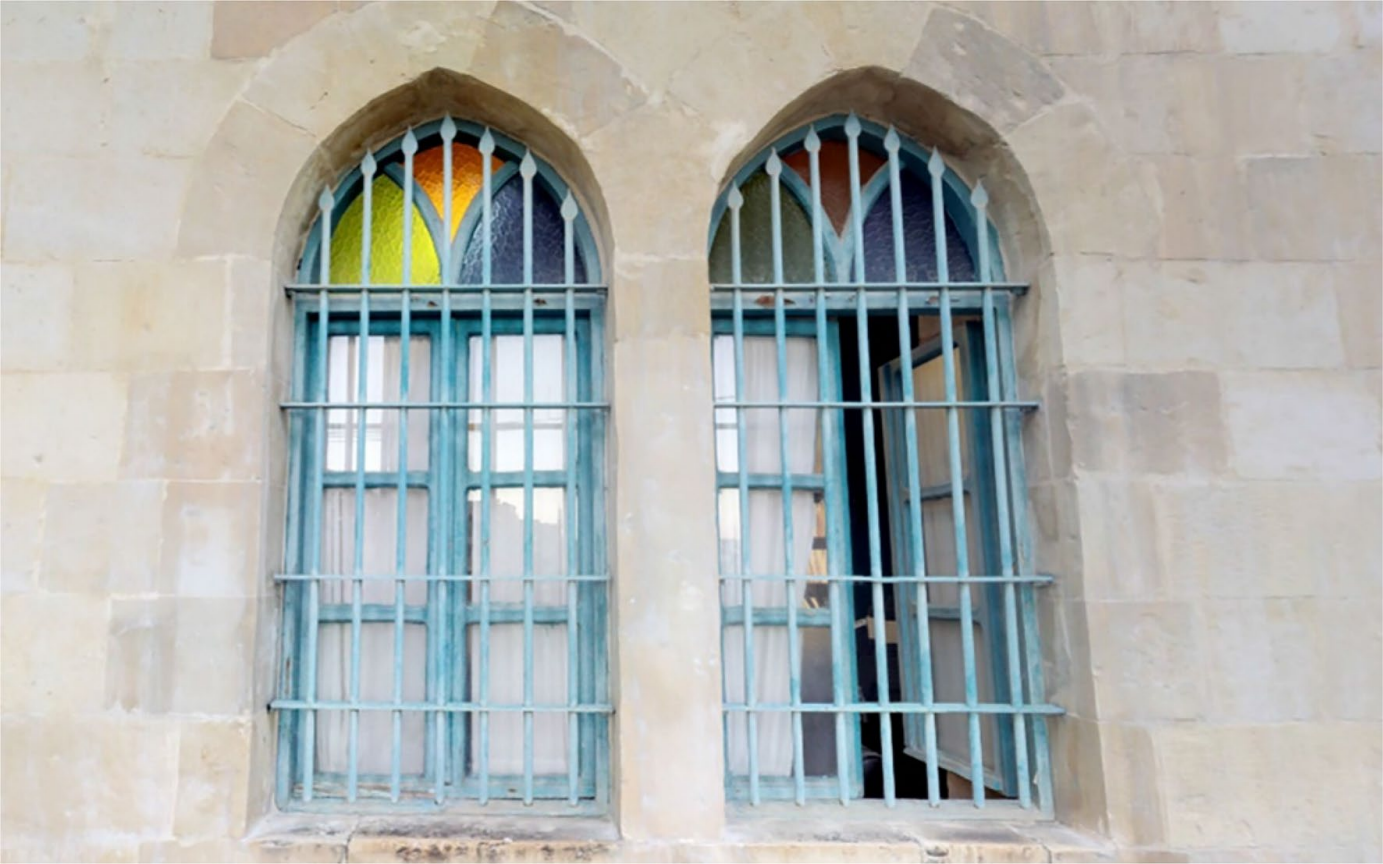
Sample of a window of the Windows Category of the Qaqish House

**Fig. 9:**
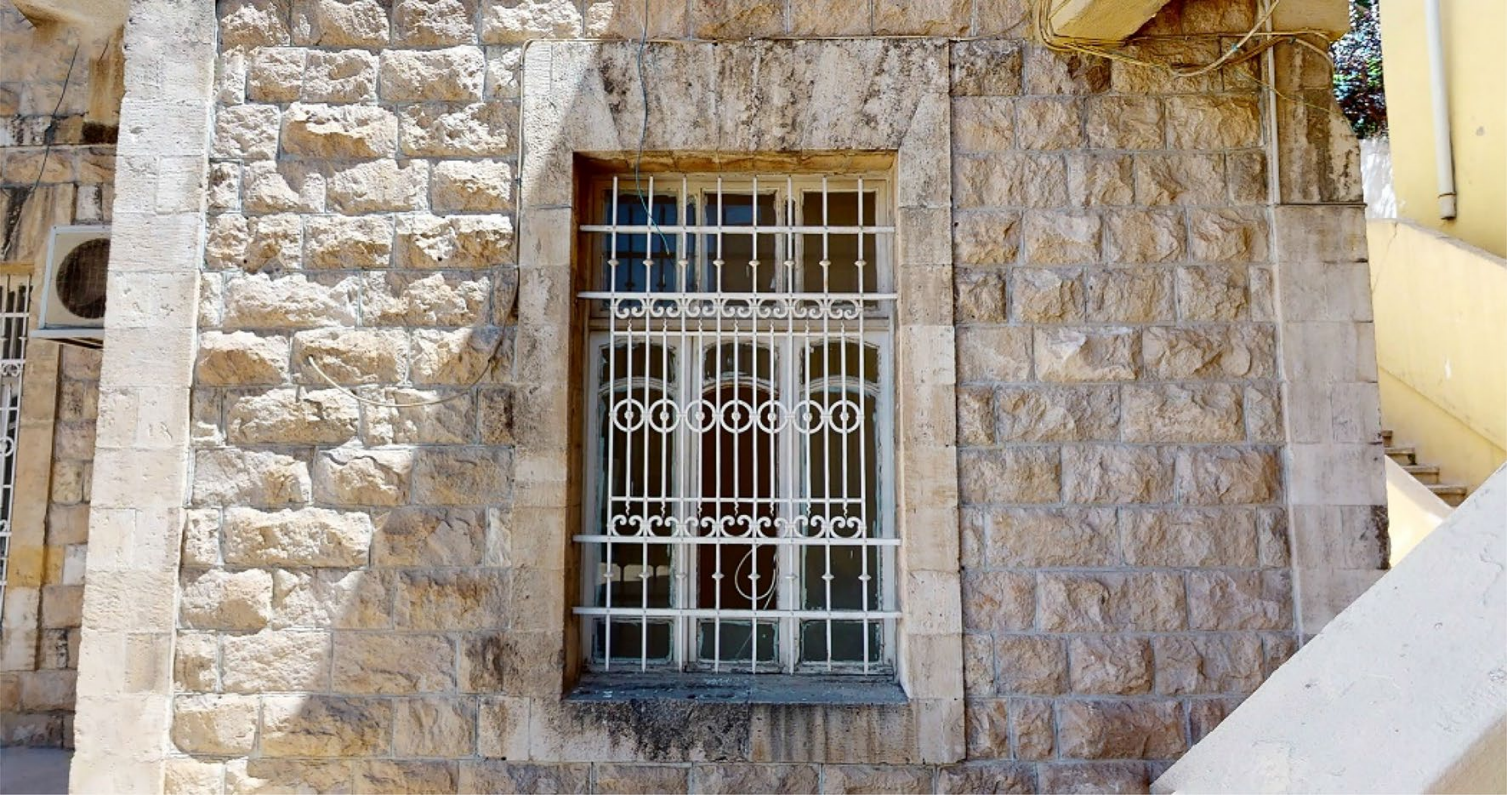
Sample of a window of the Windows Category of the House of Art

Regarding the recognition-object phase, thirty elements of interest were identified (Figs. 8, 9), which were grouped according to five categories for the House of Art: windows, doors, arches, columns, and balustrades (Table 2), and three categories for Qaqish House (Table 3): windows, doors and arches. Each element of the categories has been given an identification code and a name, indicating the storey to which they refer.

**Table 2:** Sample of the classification by category of the families composing the House of Art

Categories	Cat. identification code	Family name	Storey
Windows	3	Main double window	1/2
	4	Double window	1/2
	9	Back window 2	1/2
	12	External window	2/2
Doors	1	Main door	1/2
	7	Back door	1/2 and 2/2
	11	Main superior door	2/2
	16	Internal superior door	2/2
Columns	2	External column	1/2
	10	Superior column	2/2
Arches	20	External arch	1/2
Balustrades	14	Balustrade	2/2

**Table 3:** Sample of the classification by category of the families composing the Qaqish House

Categories	Cat. identification code	Family name	Storey
Windows	2	Internal window	2/2
	4	Superior window	2/2
	5	Arched window	1/2
	10	Window	2/2
Doors	1	Internal door	2/2
	6	Glass superior door	2/2
	7	External door	1/2
Arches	3	Internal arch 2	1/2
	8	Internal arch	1/2
	9	External arches	2/2

Since the families that have been modelled are Revit loadable families (usable in other projects through the modification of the parameters), they have been created in an external file (RFA extension), in such a way to be later loadable in the family editor and imported into any Revit project: this is only possible adding some information parameters. This means that the families that have been produced for both houses may also be used for other buildings that present similar morphological characteristics.

**Fig. 10:**
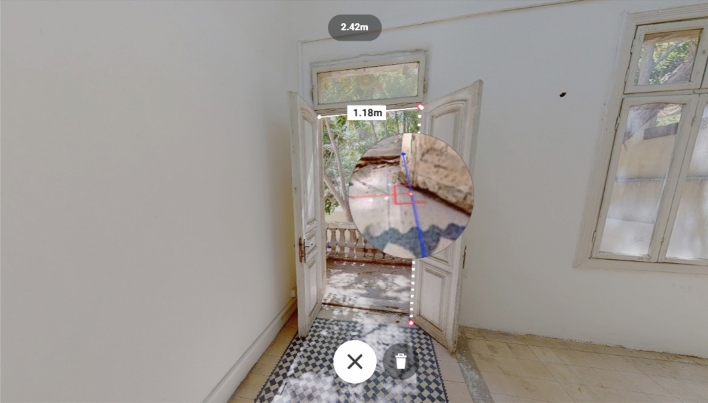
Measurement tool in the Virtual Tour of the House of Art, Amman

In the decision-making phase, the first point consisted in studying and understanding the geometry of the building, which was possible through both the point cloud and the virtual tour generated by the Leica TrueView software. Using these two tools, it was possible to measure the true size of the elements, thinking in a digital representation faithful to reality (Fig. 10). At this point, a crucial step is the definition of the Level of Development. According to the US Standards, a LOD 300 was established: in addition to quantity, size, shape, and location, geometrical elements are also linked to more in-depth non-graphical information than the previous level.

For this purpose, it was necessary to implement a Level of Accuracy to be respected. A corresponding to USIBD LOA 30 was settled. This means that deviations between the geometric model and the point cloud in the range of 5 mm and 15 mm are allowed. In the first instance of design, the elements are modelled with a simple fit. Where the deviations between model and measured data exceeded this LOA, a detailed fit is applied.

As mentioned before, the redesign phase takes place in the Revit family editor: external families are developed in.rfa format in order to be implemented in the different Jordanian architectural projects with similar geometrical characteristics. Since the point cloud cannot be inserted in this editor (in contrast to a project model), with the True View tool it was necessary to take the corresponding measurements.

**Fig. 11:**
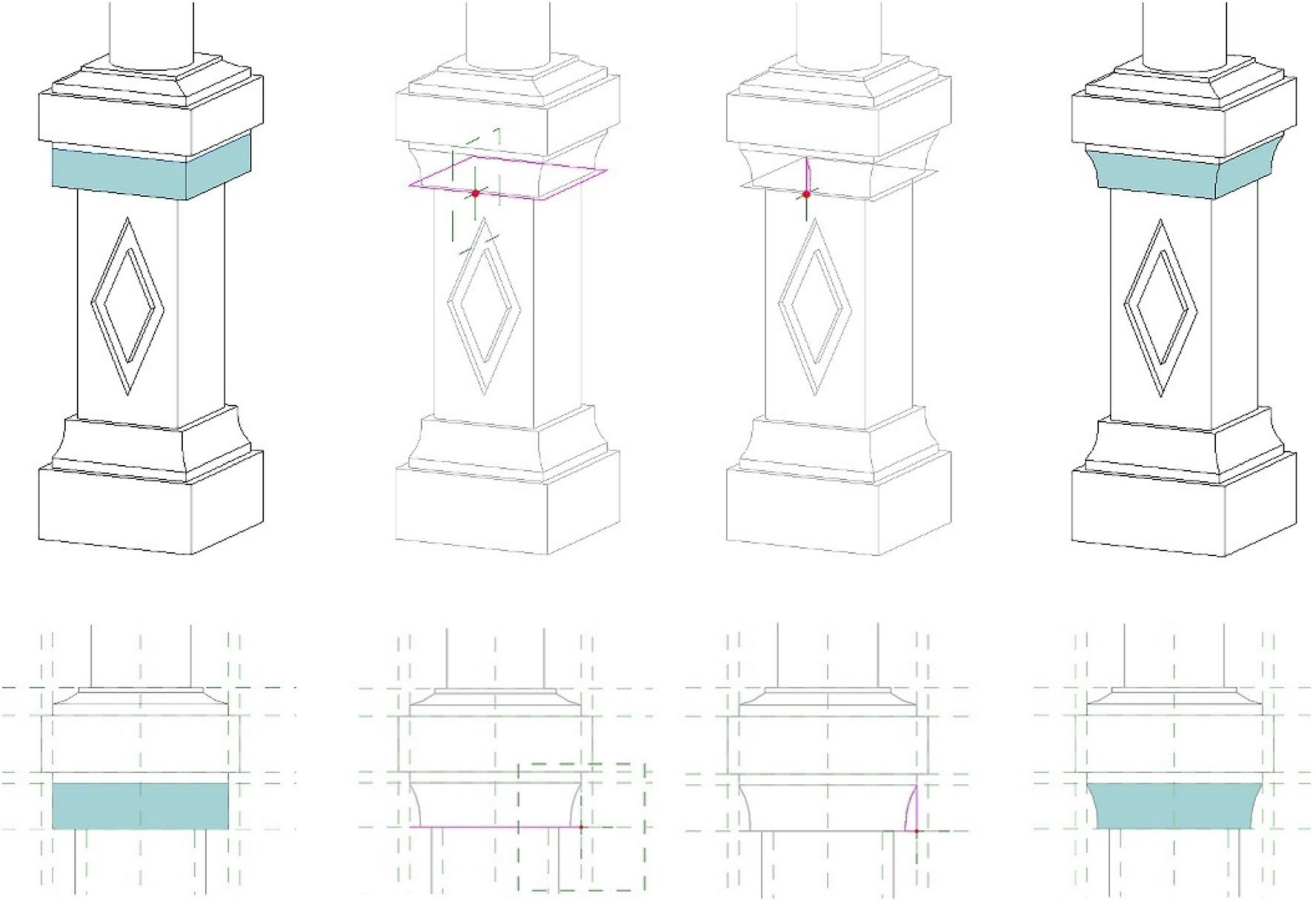
Step-by-step to create one of the sweep forms composing the column

The geometry of the element is broken down into its elementary parts, which are then modelled individually through commands like extrusion, revolve and sweep (Fig. 11), both in the form of solids and voids. In a practice case, to create the columns, the varieties of solid and void forms used are extrusion, revolve, and sweep. The shape perimeter is bound to parameters (in the form of quotas) and to reference planes (in green), so that, when the parameters related to those given reference planes are changed, the geometry moves in the desired way. All the parts of the column will depend on instance parameters, so that, in case of scale change or modulo adjustments, the column will move to fit the new dimensions.

**Fig. 12:**
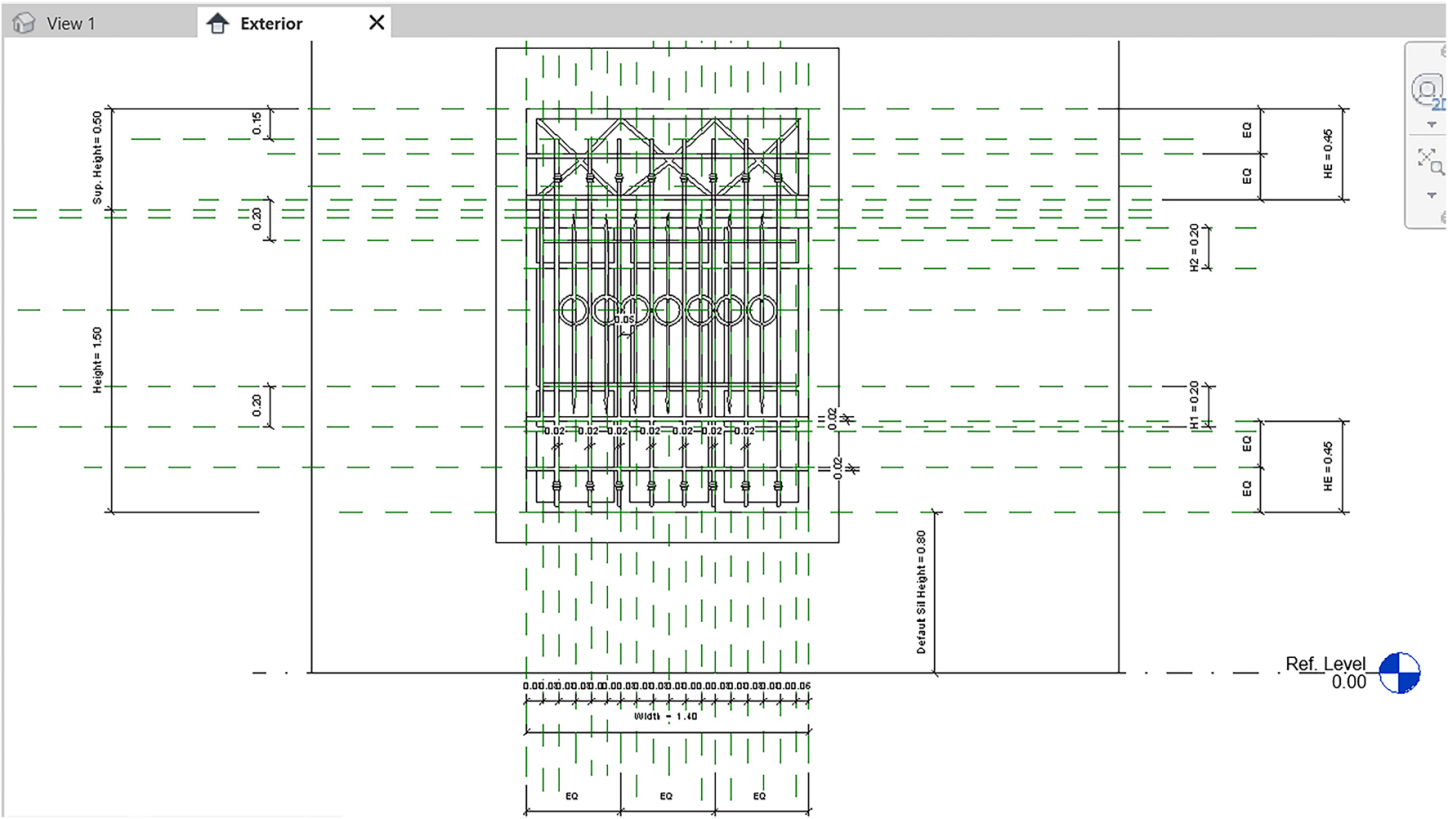
Parameters and reference planes applied to a windows family in the Qaqish House

In the case of the windows, after modelling all its parts (including sashes, frames, casings, mullions, and eventual window bars) making sure to constrain them to the reference planes, it can be tried to flex the family by editing its parameters to check if everything has been modelled following the parameters (Fig. 12). These windows must adapt to walls with different thicknesses, passing from external to internal walls, and this is where the parameters come to help.

**Fig. 13:**
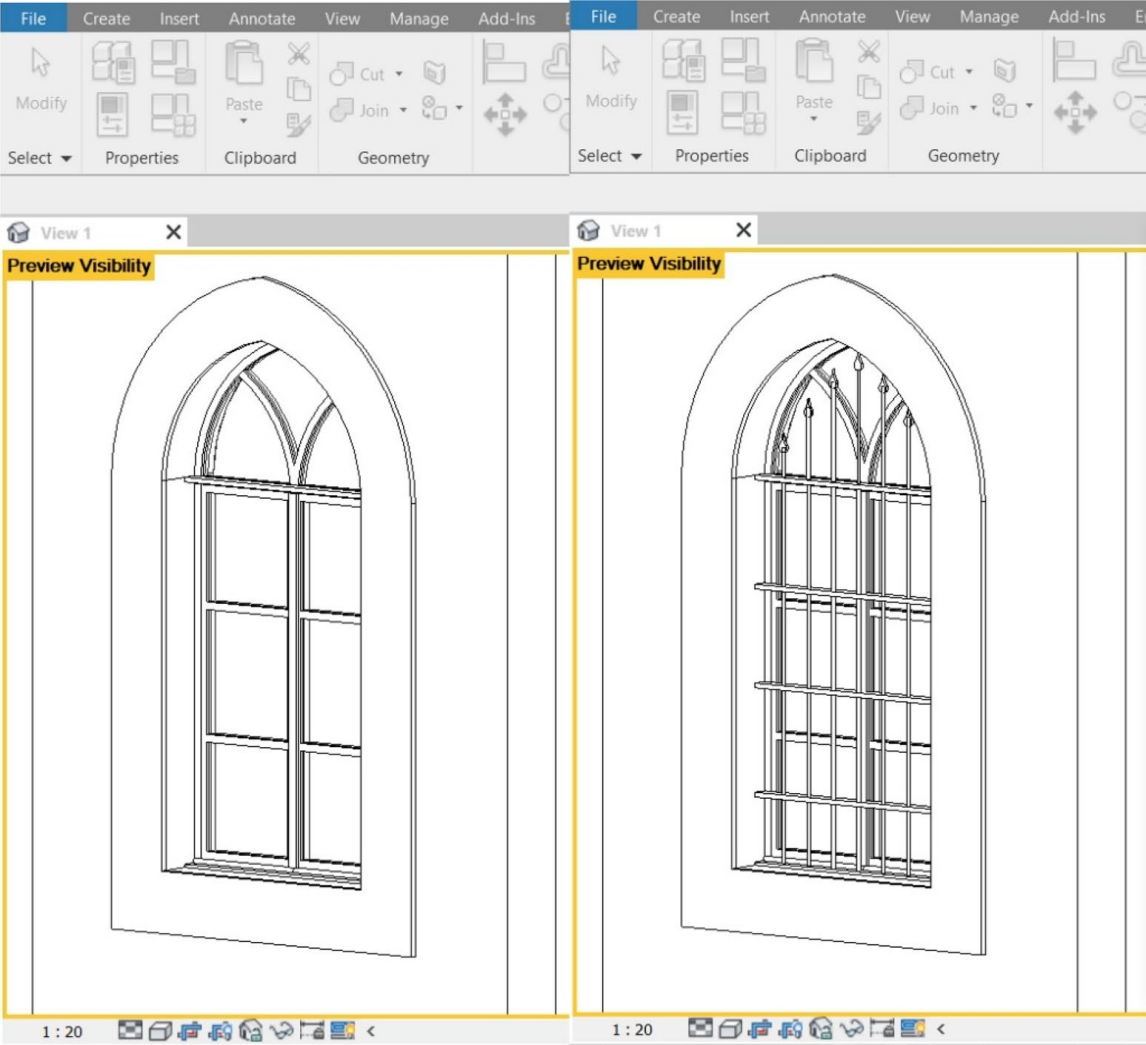
Window with window bars switched off and on in the Qaqish House model

To make the model more easily adaptable to any other project, it was necessary to create two different Family Types: one for the window itself, and another one containing the window bars (Fig. 13). In this way, it is possible to switch the visibility of the window bars off or on in case the windows of the building in which we are loading the family do not present this feature. This allows reusing the objects multiple times in one model or many different models. As previously mentioned, by modifying the parameters of a given element, the surrounding elements adapt to these changes.

**Fig. 14:**
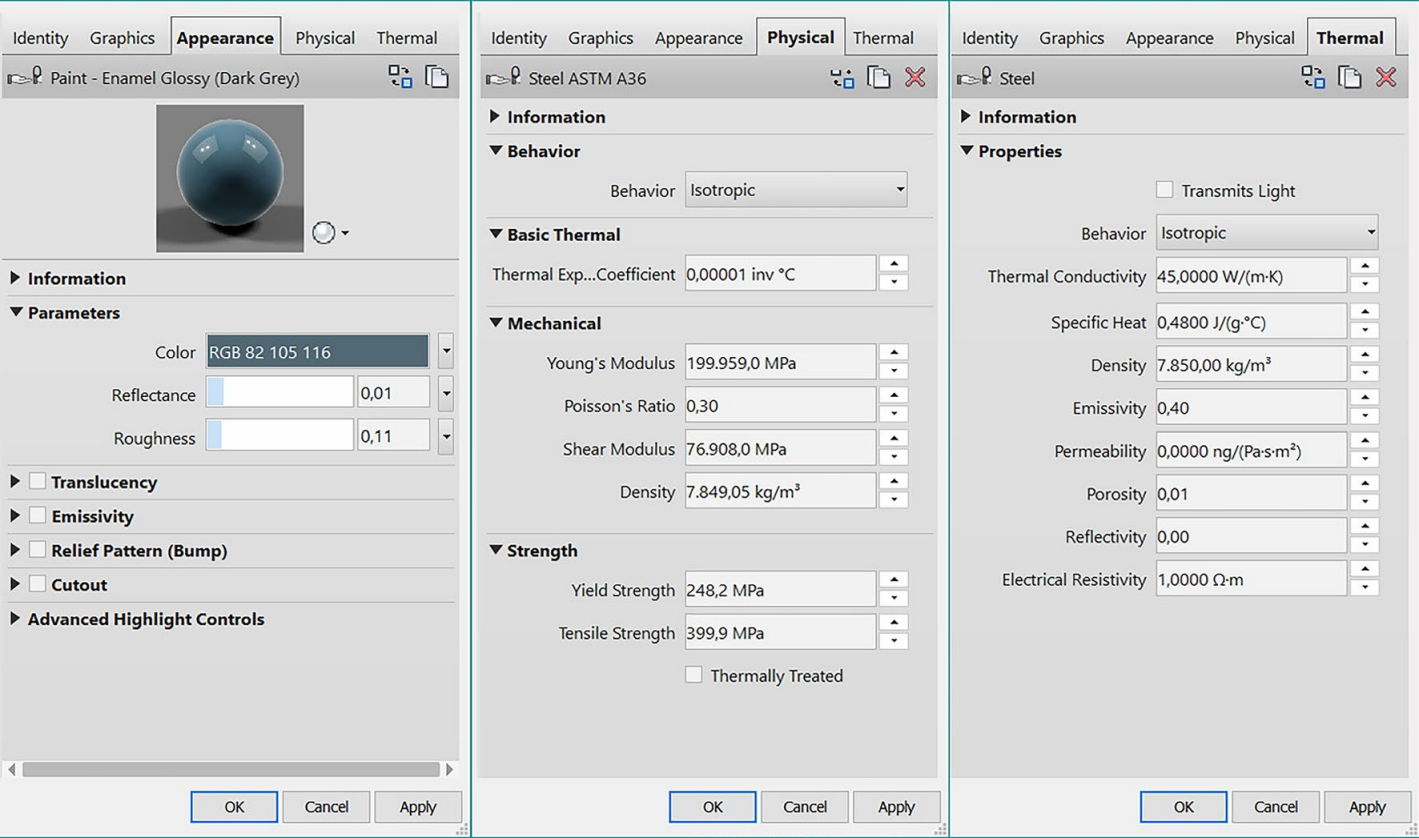
Appearance, physical properties, and thermal properties of the window bars

Once the modelling is complete, it has been necessary to introduce the building materials with their specific properties; they have been chosen from Revit Materials Browser. These have been edited (Fig. 14) according to our needs, to make them as similar as possible, in terms of aesthetics and physical and mechanical characteristics, to the materials of the House of Art.

### Creation of shared parameters

HBIM's most powerful feature is the integration of data: creating a parametric 3D model (the BIM families), every object will be linked to a table (the Types Properties table), where its parameters are shown. The main problem with these parameters is that they do not include information like historical documentation, photos, texts, condition details, or URLs, despite being largely required in the heritage field.

**Fig. 15:**
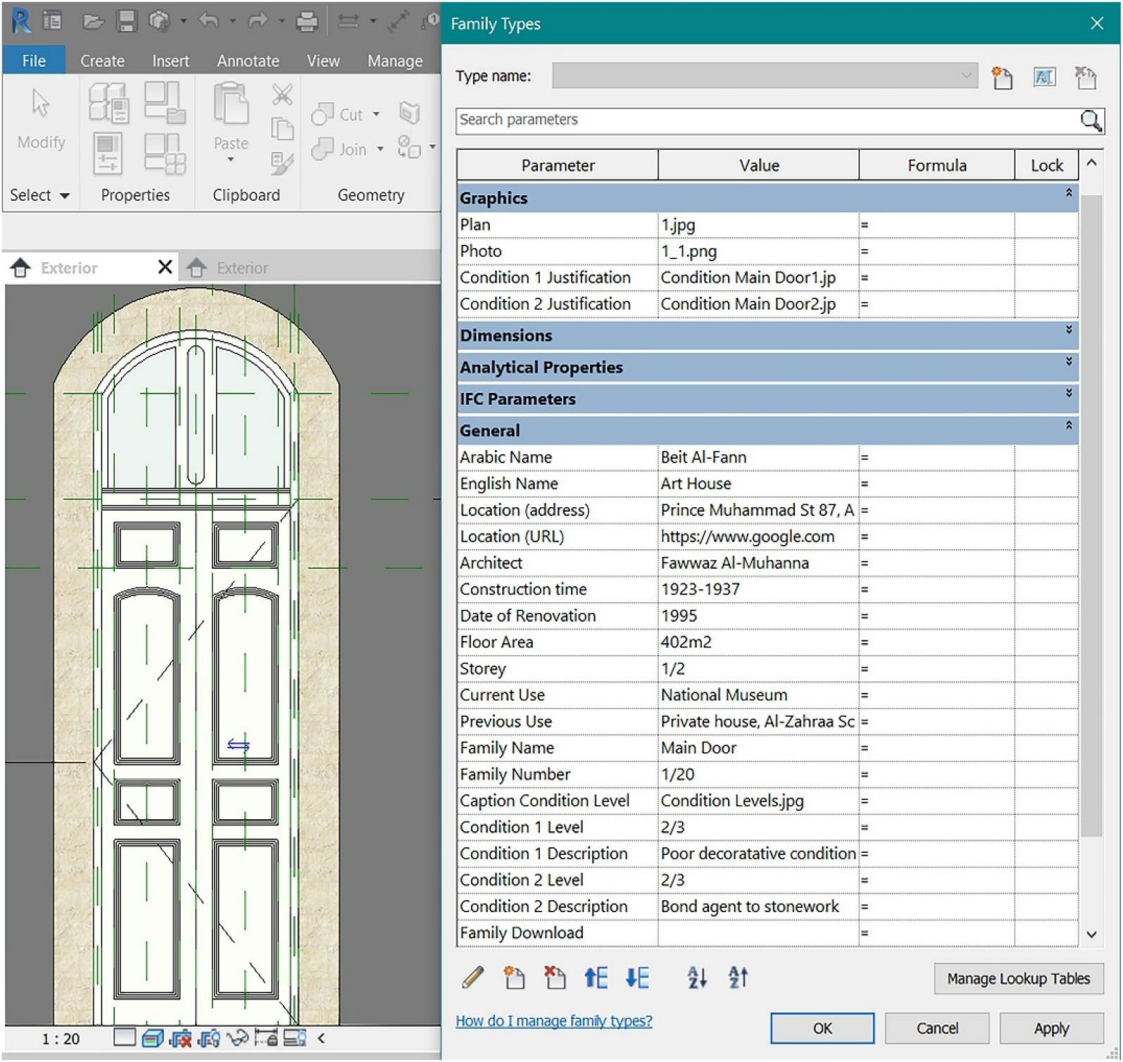
Shared Parameters assigned to the door family of the House of Art

The solution has been found in the creation of Revit Shared Parameters, which can be used in multiple families or projects and by multiple entities: to do this, Revit creates a.txt file independent of any family file or Revit project, allowing us to access the file from different families or projects (and even different devices). It has been first created the Parameter Group “House of Art” containing all the parameters/information we are interested in showing (Table 4). The parameter group will be loaded among the Family Types and the corresponding description will be added (Fig. 15).

**Table 4:** Images contained in the PBR material folder and their characteristics [28]

Colour	Roughness	Bump	Ambient occ
			
It contains the base colour input. It is a raw colour with no lighting information	It defines irregularities in the surface (white = maximum roughness)	It returns the depth and texture of the material, giving three-dimensionality	It is able to reduce unwanted reflections from ambient diffuse light

**Fig. 16:**
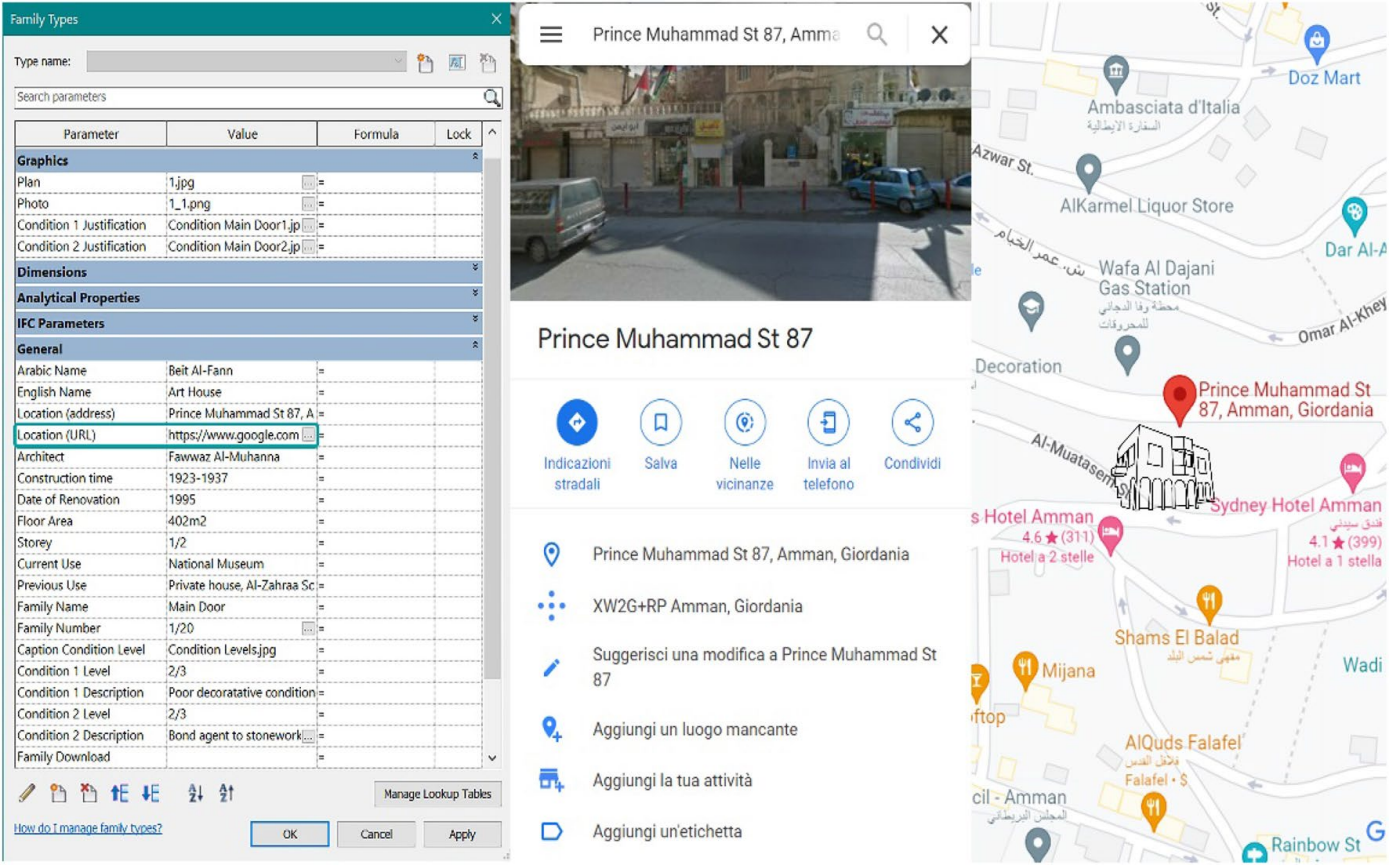
Manage icon next to the URL parameter

In this way, it is possible to store in one place all the information related to that particular Family and the building to which it belongs. In addition to the simple text parameters, there were introduced URL parameters, capable of storing information such as the Google Maps location of the houses (Fig. 16), accessible by clicking on the relevant parameter, and image parameters, relating to the position of the element in the architectural plan of the house or its state of conservation.

Once the parametric families have been created, it has been created a web portal where all the families can be uploaded, to ensure that these objects can be downloaded by every part of the world and by every practitioner operating in the conservation field and used both for buildings in Jordan (specific materials, common material degradations, local construction techniques) and for heritage buildings in general.

### Implementation of the texture

For what concerns the building stone, the Jordanian limestone, the limestone present in the Asset Browser of Revit did not respect the aesthetics of the house. For this reason, in a final modelling step, it was decided to implement the texture by using PBR materials [27], downloadable from online libraries.

PBR—Physically Based Rendering—is a rendering method that leads to higher accuracy of the representation of materials depending on how the light interacts with the surfaces on which is poured in a physically plausible way. For this case, colour, roughness, bump, and ambient occlusion maps were selected (Table 4).

**Fig. 17:**
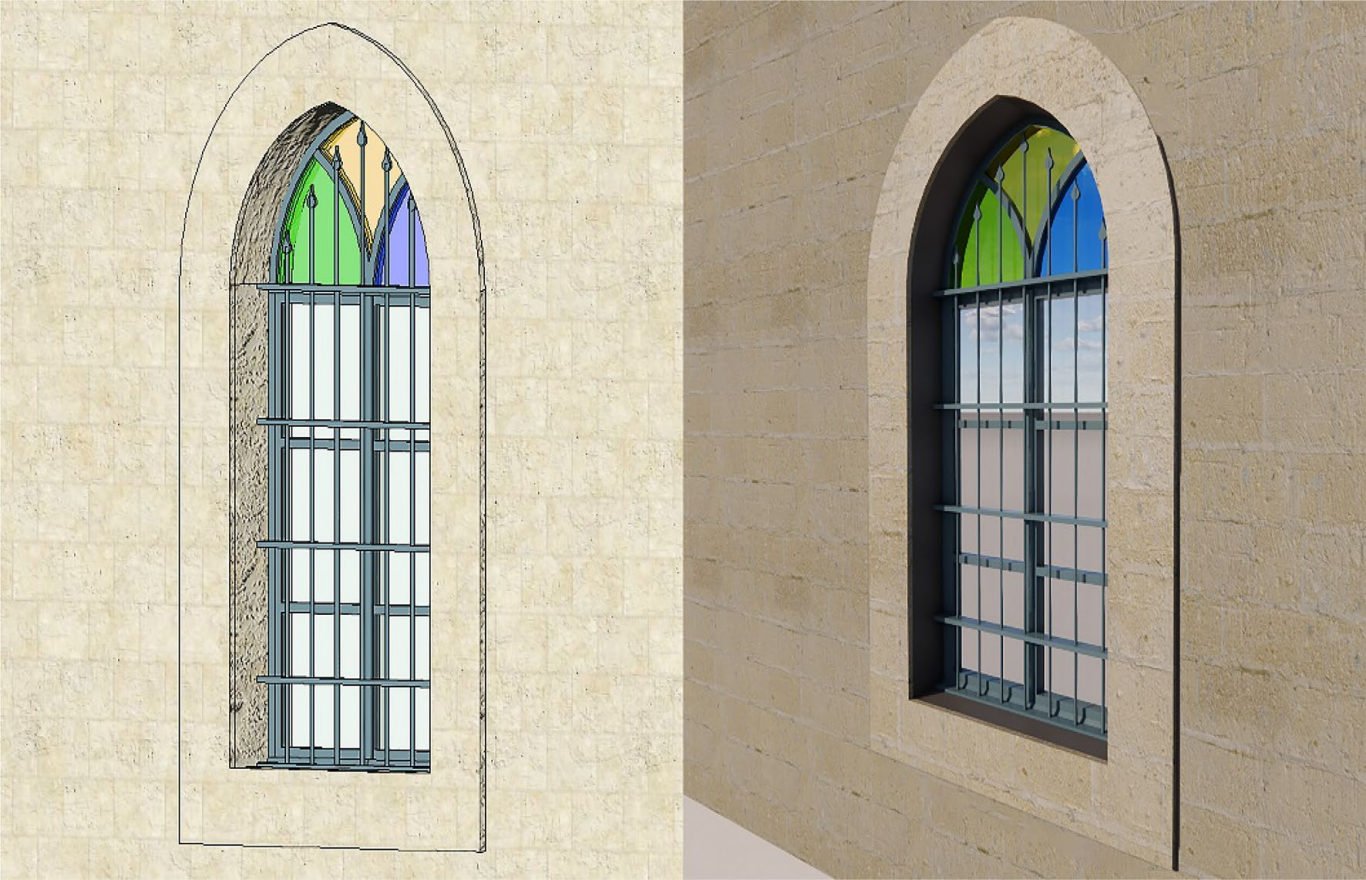
Comparison between the side view of the window of the Qaqish House visualized through Revit Realistic Visual Style and rendered on Enscape

**Fig. 18:**
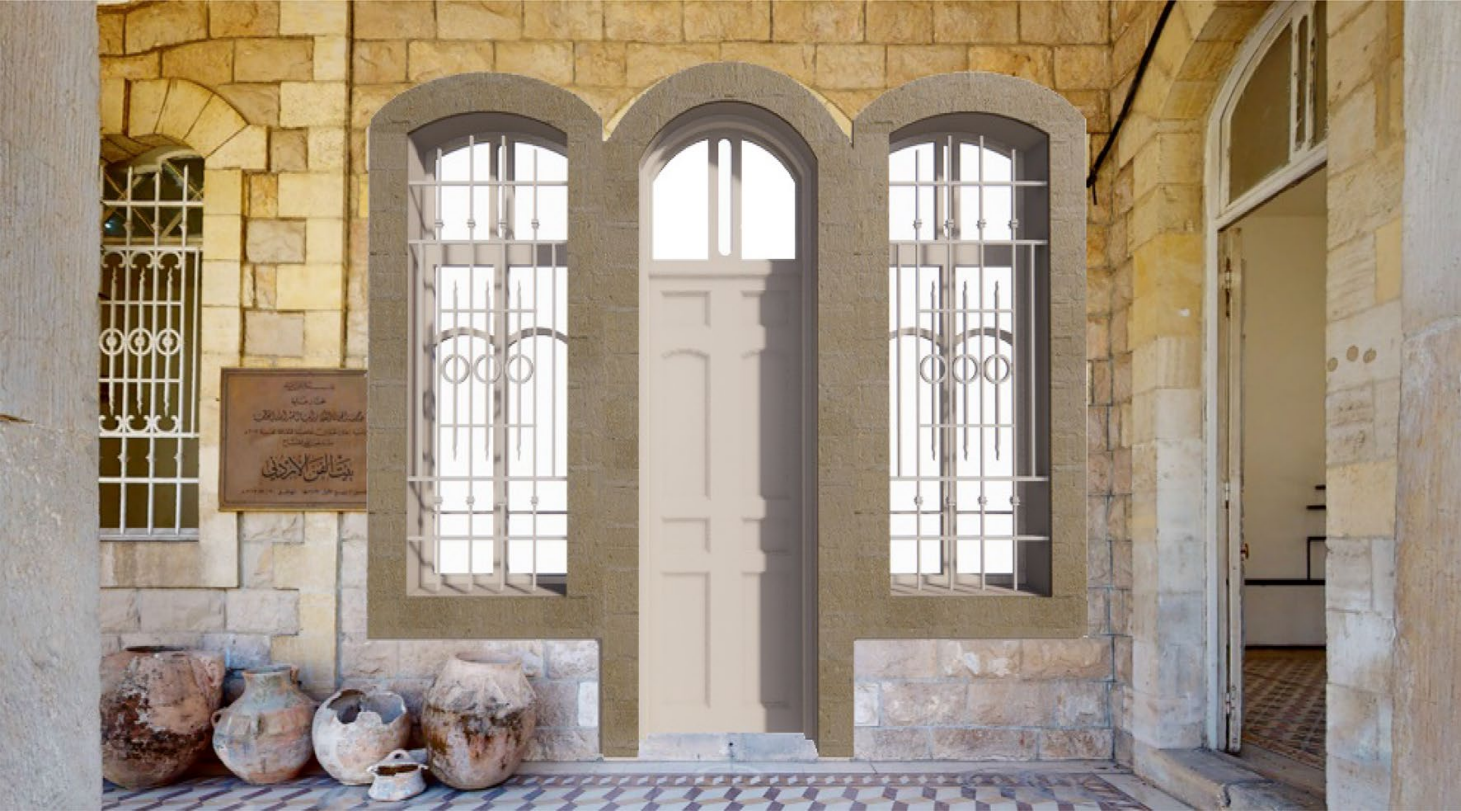
Photo insertion of the rendered main door and windows on the facade of the House of Art

When these maps are uploaded into Revit as the Appearance of the material renamed Jordanian Limestone, it is possible to render the family through a plug-in called Enscape, able to keep design and visualization all in one place, rendering a much more truthful visualization of reality than the realistic visualization of Revit (Fig. 17). From a distance, many details of the limestone can get lost, but looking closer the level of detail is clearly higher, able to guarantee the veracity of the real object (Fig. 18).

## Online 3D BIM library

**Fig. 19:**
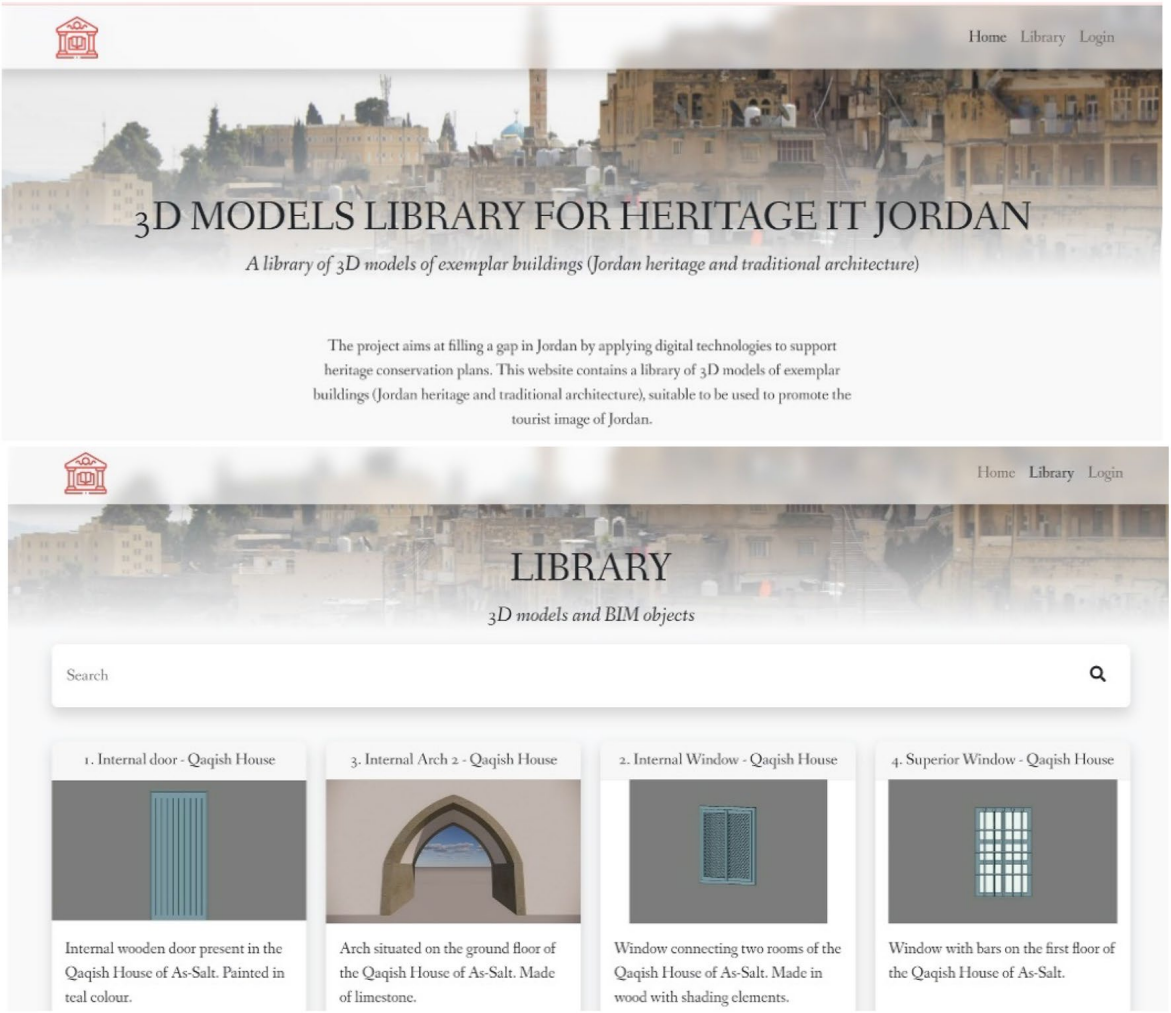
The home screen of the online BIM library, developed by the ICT University of Salerno

The online BIM library represents the culmination of this project, fulfilling the purpose that has been set at the beginning, which is the divulgation of Jordanian heritage and the promotion of the tourist image of Jordan (Fig. 19). This will be the first library of historical elements of the Jordanian built heritage, crucial because it will set a precedent for further documentation and heritage conservation of traditional cities in Jordan, MENA countries and internationally. Once all the families were completed, an online portal was created to upload them, which can be visited from the Herit-IT Jordan project website, thus ensuring that the modelled objects can be downloaded from anywhere and by any professional involved in the conservation of Jordan's heritage.

The platform was created in collaboration with the ICT centre for Cultural Heritage of the University of Salerno [29]; through this platform, the user can register and download the family most suitable for their project, answering only two simple questions that consider the location of the institution or professional that uses the families and the final use of these. He may, if necessary, upload his library of unique families, in such a way as to continue the dissemination of the Jordanian heritage. The families were loaded in the.rfa extension (belonging to all Revit loadable families) and can be downloaded in the same extension. For better compatibility with the software of the other professionals, it was decided to work with the most updated version of the Revit software.

On the platform there will be four types of users:

- The unauthenticated user can browse the BIM library of the models that have been uploaded with public visibility, but he cannot download these elements;
- The registered user can browse the BIM library and download the files attached to it;
- The editor user is a registered user, but he has the ability to upload content on the platform and edit them; and
- The administrator user is able to edit any type of content, and manage users. This user is managed by the University of Salerno.

It should also be noted that this methodology allows the work to be conducted entirely remotely, helping to continue heritage conservation even in times of pandemic or in situations that are difficult to access. The HBIM models generated according to the proposed methodology can, in the future, be used to support heritage conservation interventions, ensuring both an updated record of past interventions and the planning of future interventions.

## Final outputs and discussion

The present research aims to use an HBIM approach for the conservation of Jordanian architectural heritage, through the generation of a library of architectural parametric elements starting from their survey, accessible by every practitioner operating in the AECO (Architectural, Engineering, Constructions and Operations) sector in Jordan and the rest of the Arabic world. This main platform of the Herit-IT Jordan project (https://herititjordan.com/) and related library (https://hitj.centroictbc.unisa.it/) include a range of digital outcomes for the two pilot heritage buildings of the Jordanian House of Art in Amman and Qaqish House in As-Salt, whose potential applications include (see Table 5).

**Table 5:** Digital outcomes from the Herit-IT Jordan project website and library and potential applications

Digital outcomes	Potential applications
Virtual tours	Tourist promotion, including e-tourism enabling e-visits during a pandemic, and real estate promotion. Opportunity to include multiple layers of information, including semantic data and storytelling documenting intangible heritage values
Point clouds	Accurate documentation of the state of the building, extremely useful in case of material losses to plan for the intervention of repair
H-BIM model of the building	Extraction of 2D architectural drawings, including floorplans, sections, and elevations, usable to support conservation interventions for the specific building and for attracting investors
H-BIM families	Systematic gathering of potentially unlimited data, including technical data related to materials, useful both for planning interventions related to the specific building, but also adaptable to similar typologies thanks to the parametric nature of the H-BIM

The development of the House of Art as a case study through the digitisation of the Jordanian characteristic features demonstrates how the process can be applied to other Jordanian and Arabic buildings, in order to enrich the documentation of traditional heritage for future generations and increase the spread of the promotion and preservation of cultural heritage. Several infographic families generated through this study are not only a 3D reproduction of the real object, but it is able to store information about its essence, capable not only of keeping physical and geometric data of the elements, but also of allowing the analysis of a morphologically complex reality.

Preservation and valorisation of cultural heritage represent a problem that already existed before the Covid-19 pandemic, which has amplified it [1]. There are many challenges that the Jordanian construction sector needs to face regarding the introduction of BIM: they may be identified as the absence of government incentives, the lack of BIM standards, the lack of BIM awareness and training as its absence in educational institutions’ curriculum, and low productivity, caused by overtime work and quality specifications [30]. HBIM has the potential to create a classification system for heritage buildings under threat and impact the application of heritage legislation and regulations. The collection, management and storing of data for digital heritage requires an awareness of the issues of time and the power structures that are involved in their collection and upon which they have a profound effect. Finance is a particularly important topic for the adoption of BIM in the Jordanian construction industry. Implementing BIM would increase costs such as the purchase of software, technical support, hardware, training, and services. Importance is also given to the human factor which affects the adoption of BIM in Jordan, such as the shortages of skilled and trained staff, the lack of interest and demand, dependence on unskilled staff in the construction sector, and lack of awareness. Communication represents another latent barrier to the development of BIM in Jordan. This includes weaknesses in communication between project parties, the lack of a legal framework for BIM, the approval system for new projects and traditional work processes [31]. Several surveys and interviews have been conducted during the years, studying the current level of BIM knowledge and experience in Jordan. One of them, conducted by the *Al-Ahliyya Amman University* between 2016 and 2017, was conducted by collecting data among Jordanian architects, engineers, contractors (AEC), and other industry respondents; it shows that only 5% of the AEC companies and organizations are using Autodesk Revit (the most popular BIM software), but without having broad expertise in BIM competences. Furthermore, only big AEC companies deal with BIM on a large scale, whilst small AEC companies do not seem to want to take a step forward into the BIM technology, not being aware of BIM benefits. Another reason behind the little use of BIM in Jordan is the cost of the software, hardware, and training that is necessary to adopt the BIM technology [32].

As a result, the digitisation of cultural heritage gives the means to be able to link society and architecture digitally: this approach allows the realisation of the asset even at a distance, through the creation of virtual tours to make the spaces accessible for everyone, especially the non-technical sector. These alternative ways to visit these assets should not be considered as an alternative way to experience the site, but only as a promotional tool useful to widen the way to access cultural heritage, mostly in times of impossibility, such as during the pandemic. Jordanian government should, then, encourage BIM implementation in construction projects at least on public works, offering incentives for BIM-based projects that can guarantee a positive outcome. Local standards for the BIM industry should also be set by the government, from a management point of view. This platform was created to that has the potential to become a single point of entry for all the digital data concerning heritage and to enable unique knowledge-sharing instruments for Jordanian planners, restorers, conservationists, citizens, and developers. In fact, in the Herit-IT Jordan project, these parametric 3D models will come with a table containing their parameters and characteristics creating a library of specific smart objects accessible to all users [33]. By creating a library of 3D parametric models of elements typical of the traditional Jordanian architecture with BIM methodology, Jordan can acquire attractiveness on the part of tourists and art lovers, starting a chain reaction to get even more digital documentation and heritage conservation of the most historic cities in Jordan and, in general, in the Arabic world.

Future uses and perspectives to expand this work to have the possibility to cover other buildings in Jordan, to create a complete documentation system, leading to a different level of knowledge of the architectural object, its story and its characteristics, and this will only be possible through an accurate digital survey and digital model. Careful attention should be given to surveying and reproducing in modelling software the historic heritage, because of its unique characteristics, materials, and construction techniques which make it non-reproducible. In the future, the BIM models can also be used for the management of the plans of heritage cities, boosting the tourist attractiveness of Jordanian cities, and helping in decision-making for conservation interventions. It would also be possible to keep an updated record of the maintenance logs, enhance the facility management of the building and plan intervention in the short, medium, and long term through a PPM (Planned Preventative Maintenance). The application of this management model could revolutionize the way the management and maintenance of historical heritage are seen, not only allowing reorganisation and structure of the entire Jordanian historical heritage, but also ensuring constant and up-to-date maintenance, thereby preventing its degradation. Also, the ability to rely on a detailed parametric model allows any type of analysis without implying constant inspections of the element, providing a rapid and effective decision-making process. Moreover, the system could be linked with fire systems and security systems to protect these buildings.

## Conclusion

The present work contributes to increasing the scope of the library of construction components and aims to support the work on HBIM projects. Creation of a BIM model representing a Historic or Heritage Building can be seen as an important platform for collaboration; however, in order to achieve this, the model must include all the information required by specialised professionals. For a rigorously accurate representation of the architectural geometry and the construction solutions adopted in historic buildings, the libraries of parametric objects available in the most used BIM-based modelling systems are very limited.

The geometric complexity and uniqueness of architectural cultural heritage, together with the phenomena of ageing and deterioration that can alter the material, make modelling particularly complex. To address this issue, the project has made use of specific families parameterised in the corresponding editor, in order to guarantee maximum correspondence and precision in the digitisation. However, after an exhaustive analysis of the state of the art, and various tests and evaluations carried out by the team, it can be concluded that the results in terms of geometric accuracy and parameter settings can be optimised by applying artificial intelligence (AI) algorithms. Dynamo becomes a more feasible tool in terms of cost, staff training and time to optimise 3D modelling and application of parameters enabling the use of BIM objects in multi-purpose scenarios, where Jordanian architecture is a clear example: while presenting the common architectural construction rules, the basis shapes can be subject to variations depending on the unique architectural asset.

In conclusion, the library of BIM objects can be managed by those representing the entire construction industry and anyone who may be interested in the conservation of Heritage, because each object will carry important information for the correct conservation of that element. In the case of the Herit-IT Jordan project, the principal stakeholders for the pilot case are represented by The Greater Amman Municipality, Jordan Tourism Board, The Jordan Museum and other stakeholders as designers, companies, professional organisations, and universities with the ambition to extend this “network” in the entire world. With this goal, it will be essential that research groups raise awareness of local needs and document historical and architectural heritage by sensitising and training the local community and professionals in the use of digital technologies.

## Data Availability

The datasets used and/or analysed during the current study are available from the corresponding author on reasonable request.

## References

[1] Trillo C, Aburamadan R, Mubaideen S, Salameen D, Makore C (2020). Towards a systematic approach to digital technologies for heritage conservation. Insights from Jordan. Preserv Digit Technol Cult.

[2] Dezen-Kempter E (2014). Diálogos Digitais: Integração Entre Dados Documentais em Sistemas de Informação Baseados no Modelo para Conservação do Património Arquitectónico. Blucher Des Proc.

[3] Trillo C, Aburamadan R, Udeaja C, Moustaka A, Baffour KG, Makore CN, Bevilacqua C, Calabrò F, Della Spina L (2020). Enhancing heritage and traditional architecture conservation through digital technologies. Developing a digital conservation handbook for As-Salt, Jordan. New metropolitan perspectives: knowledge dynamics, innovation-driven policies towards the territories’ attractiveness.

[4] Murphy M, Meegan E, Keenaghan G, Chenaux A, Corns A, Fai S, Chow L, Zheng Y, Dore C, Scandurra S, Tierney A, Diara F, Rinaudo F, Prizeman O (2021). Shape grammar libraries of European classical architectural elements for historic BIM. Int Arch Photogramm Remote Sens Spat Inf Sci.

[5] Oostwegel LJN, Jaud Š, Muhič S, Rebec KM (2022). Digitalization of culturally significant buildings: ensuring high-quality data exchanges in the heritage domain using OpenBIM. Herit Sci.

[6] Herit-IT Jordan. Herit-IT Jordan. https://herititjordan.com/. Accessed Aug 2022.

[7] Oreni D, Brumana R, Georgopoulos A, Cuca B (2014). HBIM library objects for conservation and management of built heritage. Int J Herit Digit Era.

[8] Turrina A, Attico D.The reconstruction of a dynamic inventory model toward shared HBIM libraries for vaulted systems. In: international event 9th ARQUEOLÓGICA 2.0 & 3rd GEORES, Spain; 2021.

[9] Bagnolo V, Argiolas R, Cuccu A (2019). Int Arch Photogramm Remote Sens Spat Inf Sci.

[10] Kontoudaki A, Georgopoulos A (2022). Int Arch Photogramm Remote Sens Spat Inf Sci.

[11] Logothetis S, Karachaliou E, Valari E, Stylianidis E (2018). Open source cloud-based technologies for BIM. Int Arch Photogramm Remote Sens Spat Inf Sci.

[12] Diara F, Rinaudo F (2021). ARK-BIM: open-source cloud-based hbim platform. Appl Sci.

[13] Ababneh A (2016). Heritage management and interpretation: challenges to heritage site-based values, reflections from the heritage site of Umm Qais, Jordan. Archaeologies.

[14] UNESCO. UNESCO. World heritage convention. https://whc.unesco.org/en/list/689/. Accessed Aug 2022.

[15] Ali N. Beit Al-Fann: an icon of Amman’s architectural identity. 2021. https://www.jordannews.jo/Section-123/Property/Beit-Al-Fann-An-icon-of-Amman-s-architectural-identity-3326. Accessed Dec 2022.

[16] Herit-IT Project. Beit Al-Fann, House of Art, Amman. https://herititjordan.com/amman/. Accessed Aug 2022.

[17] Jordan Engineers Association. Amman building & urban planning regulation of 2011. ULTIMIT Advanced Turnkey Solutions, Amman; 2011.

[18] Al-Dustor, Sameh K. The Jordanian Art House: a national archive for the launch of the artistic and cultural movement in Jordan. 2007. https://www.addustour.com/articles/480014?fbclid=IwAR3ZRk37Lr0938-l-DE1murrTEiRa4LzFEI2kiFEY-gnzaXxZ1xE_t7k4rE. Accessed Dec 2022.

[19] Aldeek ZAO, Labin AMJE (2017). The design elements and building techniques at the traditional Jordanian dwellings. Int Rev Eng Comput Sci.

[20] Kuwait News Agency. House of Art: a civilized container that preserves the memory of generations and displays the authentic Jordanian heritage. 2003. https://www.kuna.net.kw/ArticlePrintPage.aspx?id=1388681&language=ar. Accessed Dec 2022.

[21] Herit-IT Jordan. Qaqish House, As-Salt. https://herititjordan.com/as-salt/. Accessed Dec 2022.

[22] Universes in Universe. As-Salt is a new World Heritage Site. 2021. https://universes.art/en/art-destinations/jordan/as-salt#c57663. Accessed Aug 2022.

[23] Balletti C, Bertellini B, Gottardi C, Guerra F (2021). Geomatics techniques for the enhancement and preservation of cultural heritage. Int Arch Photogramm Remote Sens Spatial Inf Sci.

[24] Hassani F (2015). Documentation of cultural heritage. Techniques, potentials and constraints. Int Arch Photogramm Remote Sens Spat Inf Sci.

[25] CAD Savvy Consulting. Leica BLK360. 2021. https://cadsavvy.ca/Details?prodId=210. Accessed Dec 2022.

[26] Santamaría J, Cordón O, Damas S (2011). A comparative study of state-of-the-art evolutionary image registration methods for 3D modeling. Comput Vis Image Underst.

[27] Vortex Studio. Physically based rendering (PBR) materials. 2020. https://www.cm-labs.com/vortexstudiodocumentation/Vortex_User_Documentation/Content/Editor/editor_pbr.html. Accessed Dec 2022.

[28] Pluralsight. Elliminate texture confusion: bump, normal and displacement maps. 2014. https://www.pluralsight.com/blog/film-games/bump-normal-and-displacement-maps. Accessed Aug 2022.

[29] Centro ICT per i Beni Culturali, Università degli Studi di Salerno. 3D models library for heritage IT Jordan. 2021. https://hitj.centroictbc.unisa.it/#. Accessed Aug 2022.

[30] Al-Btoush MAKA, Al-Btoosh AA (2019). BIM adoption strategies—the case of Jordan. Int J Civil Eng Technol.

[31] Alshdiefat A (2017). Developing an assessment model for the adoption of building information modelling to reduce the cost of change orders in the jordanian construction industry.

[32] Matarneh RT, Hamed SA (2017). Exploring the adoption of building information modeling (BIM) in the Jordanian Construction Industry. J Archit Eng Technol.

[33] Pocobelli DP, Boehm J, Bryan P, Still J, Grau-Bové J (2018). BIM for heritage science: a review. Herit Sci.

